# ADP-ribosyl transferase activity and gamma radiation cytotoxicity of *Pseudomonas aeruginosa* exotoxin A

**DOI:** 10.1186/s13568-021-01332-3

**Published:** 2021-12-22

**Authors:** Radwa N. Morgan, Sarra E. Saleh, Khaled M. Aboshanab, Hala A. Farrag

**Affiliations:** 1 National Centre for Radiation Research and Technology (NCRRT), Drug Radiation Research Department, Egyptian Atomic Energy Authority (EAEA), Ahmed El-Zomor Street, Nasr city, Cairo, 11787 Egypt; 2 Microbiology and Immunology Department, Faculty of Pharmacy, Ain Shams University, African union organization Street, Abbassia, Cairo, 11566 Egypt

**Keywords:** Exotoxin A, ADP ribosyl transferase activity, NBAG, Gamma radiation, Cytotoxicity, HPLC

## Abstract

This work explores the ADP-ribosyltransferase activity of *Pseudomonas (P.) aeruginosa* exotoxin A using the guanyl hydrazone derivative, nitrobenzylidine aminoguanidine (NBAG) and the impact of gamma radiation on its efficacy. Unlike the conventional detection methods, NBAG was used as the acceptor of ADP ribose moiety instead of wheat germ extract elongation factor 2. Exotoxin A was extracted from *P. aeruginosa* clinical isolates and screened for *toxA* gene using standard PCR. NBAG was synthesized using aminoguanidine bicarbonate and 4-nitrobenzaldehyde and its identity has been confirmed by UV, FTIR, Mass and ^13^C-NMR spectroscopy. The ADP-ribosyl transferase activity of exotoxin A on NBAG in the presence of Nicotinamide adenine dinucleotide (NAD^+^) was recorded using UV spectroscopy and HPLC. In vitro ADP-ribosyl transferase activity of exotoxin A protein extract was also explored by monitoring its cytotoxicity on *Hep-2* cells using sulforhodamine B cytotoxicity assay. Bacterial broths were irradiated at 5, 10, 15, 24 Gy and exotoxin A protein extract activity were assessed post exposure. Exotoxin A extract exerted an ADP-ribosyltransferase ability which was depicted by the appearance of a new ʎmax after the addition of exotoxin A to NBAG/NAD^+^ mixture, fragmentation of NAD^+^ and development of new peaks in HPLC chromatograms. Intracellular enzyme activity was confirmed by the prominent cytotoxic effects of exotoxin A extract on cultured cells. In conclusion, the activity of Exotoxin A can be monitored via its ADP-ribosyltransferase activity and low doses of gamma radiation reduced its activity. Therefore, coupling radiotherapy with exotoxin A in cancer therapy should be carefully monitored.

**Supplementary Information:**

The online version contains supplementary material available at 10.1186/s13568-021-01332-3.

## Key points

- NBAG was easily synthesized and served as good acceptor of ADP ribose moiety.
- Exotoxin A exhibited an ADP-ribosyltransferase activity on NBAG.
- Low doses gamma radiation affects the enzyme activity adversely.

## Introduction

Pathogenic *Pseudomonas (P.) aeruginosa* possess virulence factors that aggravate the severity of their infections and contribute to series outcomes. Exotoxin A is the most prevalent and toxic virulence factor among pathogenic *P. aeruginosa* species that acquires ADP-ribosyltransferase activity. It is the reason behind the increased mortality rates among experimentally infected animals where a single injection of 80 ng was sufficient to induce severe liver necrosis and swelling, hemorrhage in the lungs and kidneys within 48 h of exposure (Wretlind and Pavlovskis 1981). A recent study proved the potential applications of nontoxic *P. aeruginosa* exotoxin A to facilitate the oral delivery of macromolecular therapeutics under conditions of covalent or non-covalent association (Li et al. 2021). Recently, Shadman et al. have developed monoclonal antibodies against exotoxin A as an alternative approach to control infection by this life threatening pathogen (Shadman et al. 2021).

*Pseudomonas aeruginosa* exotoxin A (PE toxin) is chromosomally encoded by *toxA* gene (ETA) which is expressed to a single chain protein made of 638 amino acids and 66 kDa Molecular weight. It belongs to two component AB toxin family that harbors multiple domains, one for cellular binding and the other responsible for the catalytic activity (Wozniak et al. 1987). It’s composed of a highly hydrophobic leader peptide of 25 amino acids followed by domain Ia, the receptor binding domain, domain II, the translocator and domain III, the ADP-ribosyl transferase catalytic moiety (Siegall et al. 1989; Michalska and Wolf 2015). To express its catalytic activity, PE toxin requires proteolytic cleavage by furins, subtilisin like cell surface serine proteases that are widely distributed in the human body (Zahaf and Schmidt 2017). The ADP-ribosylation pathway of PE toxin follows an SN1 nucleophilic substitution mechanism where it first attaches itself to NAD^+^ via active site loop L4 (aa 483–490 of domain III) (Yates and Merrill 2004). This attachment split the C–N bond between the *N*-ribose and nicotinamide forming an oxacarbeniumin intermediate at the E553 residue of the toxin. Following the splitting of the NAD^+^, PE toxin attack elongation factor 2 at diphthamide residue and the ADP ribose is attached to N3 atom of diphthamide imidazole ring. Consequently, EF-2 becomes ADP-ribosylated and inactive, halting the protein synthesis within the affected cell via their inability to elongate polypeptide chains. This induces irreversible cellular death (Armstrong et al. 2002; Jørgensen et al. 2005; Michalska and Wolf 2015). In In vitro studies, the commonest ADP-ribosyltransferase enzyme activity assay conducted includes covalent attachment of radioactive ADP-ribose obtained from (adenylate-32Pi) NAD or (adenine-U-14C) NAD to wheat germ protein. The covalent modifications are then detected by measuring the levels of released isotopes using special detectors. A new method for the detection of ADP-ribosyl transferase activity was later introduced by Soman et al. who utilized the use of guanylhydrazones derivatives as acceptors of the ADP ribose moiety post exposure to the enzymes. The guanyl hydrazones derivatives have been deployed in the detection of the ADP-ribosyltransferase activity of Cholera toxin and ribosyl-transferases retrieved from animal tissues (Soman et al. 1983).

Further, PE toxin blocked the production of antiapoptotic proteins within mouse embryo fibroblasts resulting in the activation of BAK induced apoptosis (Michalska and Wolf 2015). Such toxic effects made PE toxin desirable for the construction of imunotoxins that are used in targeted anticancer therapy. This was done by the removal of domain I and replacing it by ligand that targets specific tumor antigens (Zahaf and Schmidt 2017). For instance, PE toxin has been fused with Fv fragment of Anti-CD22 monoclonal antibody that binds to CD22 cell surface receptor expressed on malignant B cells and has been currently approved for treatment of relapsed or refractory hairy cell leukaemia under the trade name Moxetumomab pasudotox-tdfk (LUMOXITI™) (Dhillon 2018). In this study, we aimed to explore the ADP-ribosyltransferase activity for exotoxin A isolated from clinical *P.s aeruginosa* isolates using NAD^+^ and the guanyl hydrazone derivative, 4-nitrobenzylidine aminoguandine (NBAG) and the impact of gamma radiation on it. Here, NBAG acted as an acceptor of the ADP-ribose moiety from NAD^+^ instead of the conventional usage of wheat germ extract elongation factor 2 and radiolabeled NAD^+^.

## Materials and methods

### Recovery and isolation of the bacterial isolates

Forty bacterial isolates were obtained as bacterial growth on nutrient agar previously recovered from pus samples (wound infection) at bacteriology unit of an educational hospital, Cairo, Egypt. The isolates were purified on Cetrimide agar (CONDA, Spain) to confirm *Pseudomonas* species identity then sub-cultured on nutrient agar for regular cultivation and storage. A total of four isolates were subjected to 16s rRNA identification and the sequenced 16S ribosomal RNA was analyzed by MegaX software to be submitted in NCBI GenBank records, compute the average pairwise distance, and construct the phylogenetic trees (Kumar et al. 2018). The four isolates were also deposited in the Culture Collection Ain Shams University (CCASU) belonging to the World Data Centre for Microorganisms (WDCM).

### Synthesis of nitrobenzylidene aminoguanidine (NBAG)

Aminoguanidine bicarbonate (Lobechem, Durban, South Africa) and 4-nitrobenzaldehyde (Lobechem, Durban, South Africa) were the precursor chemicals for the synthesis reaction. First, 0.3 M aminoguanidine bicarbonate solution (100 ml) was prepared by dissolving 4.08 g in 1 N HCl. An equimolar solution of 4-nitrobenzaldehyde (100 ml) was formulated by dissolving 4.536 g in 70% ethanol. Aminoguanidine bicarbonate solution was then warmed to 60 °C and 4-nitrobenzaldehyde solution was added to it in a drop wise manner. The mixture was stirred using a magnetic stirrer overnight at room temperature. The precipitate was collected by freeze drying (lyophilization) and the powder was analyzed by FTIR, UV and mass spectroscopy and ^13^C-NMR-dmso_d6_ to confirm the identity of NBAG (Soman et al. 1983; Wu et al. 2019).

### FTIR and UV spectroscopic analysis of lyophilized powder

The chemical bonds in lyophilized powder were identified by Bruker Vertex 70 v vacuum FTIR spectrometer at a wavelength range 400–4000 cm^−1^. Afterwards, the lyophilized powder was dissolved in 0.1 N HCl and 0.1 N NaOH at concentrations of 39 µM and 312.5 µM, respectively. The UV absorption spectrum for both solutions was recorded over 200–800 nm at room temperature using double-beam Jasco V-670 (UV/VIS) spectrophotometer. Both IR and UV spectrums were analyzed to confirm the identity of the lyophilized powder (Soman et al. 1986).

### Mass spectroscopic analysis of lyophilized powder

The mass spectroscopic analysis was performed at Nawah Scientific Co., Egypt using Advion compact mass spectrometer (CMS) NY | USA. The lyophilized powder was dissolved in methanol then it was spotted on a TLC plate before the injection. Typical fragmentation was performed at a mass range 100 to 1200 and type ESI and APCI were applied. The analysis was done against two standards sulphadiazine (MWT = 250 g/mol) and quercetin (MWT = 302 g/mol) to ensure the quality of the analysis.

### ^13^C-NMR-dmso_d6_ spectroscopic analysis of lyophilized powder

The ^13^C-NMR was used to infer the chemical bond present in the lyophilized powder (Soman et al. 1983; Wu et al. 2019). The sample was dispersed in dmso_d6_ and analyzed by Jeol ECA 500 II NMR, Mansoura University.

### Detection of exotoxin A (*tox*A gene) among the recovered *P. aeruginosa* isolates

The primers for the detection of exotoxin A, *tox*A gene, were designed using primer blast tool NCBI. The primers were designed to amplify the ADP ribosyl transferase catalytic domain moiety. The sequences for the forward and reverse primer were (5′-GGCTATGTGTTCGTCGGCTA-3′) and (5′-CAGCCGAGAATGGTCTCCAG-3′), respectively. Colony PCR was performed to detect the presence of *tox*A gene among the recovered isolates. In a 100 µl Eppendorf, a single colony from an overnight bacterial growth on nutrient agar was suspended in 100 µl deionized water. The bacterial suspension was heated at 99 °C for 6 min. The PCR reaction tube was prepared as follows; 6.5 µl deionized water, 12.5 µl of master mix (Cosmo PCR Red M. Mix, Willowfort, Jena, Germany), 1 µl of 2.5 µM forward primer, 1 µl of 2.5 µM reverse primer and 2 µl of the preheated bacterial suspension. The program used for the detection of *tox*A gene was: Denaturation at 95 °C for 10 min, followed by 30 cycles of denaturation at 94 °C for 30 s, annealing at 59.4 °C for 30 s, and extension at 72 °C for 30 s, then the reaction was terminated by a final extension round at 72 °C for 7 min. Afterwards, the produced amplicons were separated by gel electrophoresis and examined by UV transilluminator. The agarose gel was prepared by dissolving 0.72 g molecular grade agarose (bioline) in 60 ml Tris borate EDTA buffer (pH 7.5–8) and 5 µl ethidium bromide was added for DNA band visualization. When the gel solidified, the PCR products were loaded against 100 bp DNA ladder (Berus 100 bp DNA ladder, Willowfort) and visualized by UV transilluminator at 312 nm (Dong et al. 2015; Amini et al. 2018).

### Sequencing of the PCR product

One of the PCR products was sanger sequenced at Colors Medical lab, Clinilab, Egypt, using Applied Biosystems 3500 series genetic analyzer. The sequence was blasted against European Nucleotide Archive and Uniprot protein databases using ExPasy blastx tool (https://web.expasy.org/blast/). The sequence was also submitted in NCBI GenBank database and granted an accession number. The phylogenetic trees and pairwise distances were computed using MegaX software which applies the Maximum Likelihood method and Tamura-Nei model. A bootstrap value of 5000 was set during the construction of the phylogenetic tree (Kumar et al. 2018).

### Exotoxin A extraction from *tox*A harboring *P. aeruginosa* isolates

From an overnight bacterial growth on nutrient agar, few pure colonies were inoculated into 50 ml dialyzed tryptic soy broth (CONDA, Spain) supplemented with 1% glycerol and incubated in an orbital shaker at a rate of 120 cycle per minute at 32 °C for 20 h. The cultures were transferred to sterile 50 ml falcon tubes and centrifuged at 4 °C at 7000×*g* for 45 min. The culture supernatant was refrigerated at 4 °C for few hours (around 3 h) then 3 ml of 1 M Zn acetate was added to the cooled supernatant. Upon the addition of zinc acetate, a white precipitate was formed and collected by centrifugation at 6000×*g* for 30 min. The supernatants are then discarded, and the pellet was dissolved in 0.3 M sodium citrate. The solution was then transferred to dialysis tube (cutoffs 12–14 KDa and a flat width of 32 mm (diameter 20.4 mm)) and dialyzed against 0.01 M tris buffer overnight at 4 °C. The protein in the concentrated dialysate was salted out with solid Ammonium sulphate at 60–70% saturation and kept at − 4 °C for 60 min to facilitate the protein precipitation. The protein precipitate was collected by centrifugation at 9000×*g* for 30 min then dissolved in 0.01 M tris buffer. The protein concentration was adjusted by UV spectroscopy using Bovine serum albumin (BSA) as protein standard. The absorptivity was calculated by computing the slope of the BSA serial dilutions (20, 50, 100, 250, 500, 1000, 2000 and 3000 µg/ml) against absorbance at 280 nm plot. Following the determination of the absorptivity, the protein concentration was deduced by measuring the absorbance of the solution at 280 nm and compensating in the following equation.

$${\text{concentration of protein }}\left( {\frac{{{\text{mg}}}}{{{\text{ml}}}}} \right) = \frac{{{\text{Absorbance at }}280{\text{ nm}}}}{{{\text{absorbitivity }} \times {\text{ b}}}}$$

where b is the path length of quartz cuvette in cm. The cuvette used in this experiment was of 10 mm path length (Liu et al. 1973; Simonian et al. 2006; Burgess 2009).

### Determining the ADP-ribosyl transferase activity of exotoxin A protein extract by UV spectroscopy

The ADP ribosyl transferase activity was detected by monitoring the changes in the absorbance and absorbance maxima at the uv range (200–500 nm). The reaction was initiated by incubating 150 µl of protein solution (376 µg/ml) with 200 µl of 0.01 M dithioerithritol (DTE), 200 µl of 0.002 M tris acetate (pH 8) and 400 µl sterile distilled water. The tubes were incubated for 30 min at 35 °C then 200 µl of sterile distilled water, 400 µl of 4 mM NAD, and 1.2 ml of 1 mM NBAG dissolved in 0.1 N HCl were added. The reaction tubes were shaken and incubated at 35 °C for 30 min prior to measuring the absorbance. The absorbance spectra were recorded against standard that contained the same components of the reaction tube without adding the protein solution. The absorbance maxima were determined and expected amount of ADP-ribosylated NBAG was computed according to the work conducted by Soman et al. (1983).

### Determining the ADP-ribosyl transferase activity of exotoxin A protein extract by high performance liquid chromatography (HPLC)

The absorbance spectra of exotoxin A extract only for some selected *P. aeruginosa* isolates was monitored using tris buffer as a blank. It was noted that the absorbance maxima of the toxin alone appeared at ʎmax ≈239–240 nm. The exotoxin A toxin extract was then added to NBAG (dissolved in 0.1 N HCl and preincubated with DTE) and NAD^+^ mixture and the reaction was monitored by observing the changes in the absorption spectra against a blank tube containing the same component of reaction tube but lacking the toxin extract (NBAG, DTE, NAD^+^, tris acetate and tris buffer). The purpose of the blank is to cancel any interference from the reaction components in produced spectrum. ADP ribosyl transferase activity was detected by HPLC. The reaction tube contained 0.2 M tris acetate (pH 8), 0.02 M DTE, 4 mM NAD, 300 µg/ml of exotoxin A protein extract and 10 mM NBAG. The reaction was terminated 3 h after exposure by the addition of 10% trichloroacetic acid (TCA). The precipitate formed during reaction was either collected and analyzed by FTIR or dissolved in absolute methanol and injected in HPLC. The mixture was analyzed using Shimadzu LabSolutions High performance liquid chromatography with C18 column; 4.6 × 250 mm, with 5 µm particle size at Faculty of Pharmacy, Ain Shams University. The mode of elution was gradient and mobile phase used was methanol. Flow rate applied was 0.2 ml/min at ambient temperature and the absorbance was measured at 301 nm. The standard used contained the same components of the reaction tube without the addition of protein extract (Soman et al. 1983; Kupiec 2004). The concentration of the ADP ribosylated product was computed as follows.

$${\text{Conc}}.{\text{ of unknown}} = \frac{{\text{Area of unknown}}}{{\text{Area of known}}}{ } \times {\text{ Conc}}.{\text{ of known}}$$

### Cytotoxicity of exotoxin A protein extract using sulforhodamine B (SRB) assay

Head and neck cancer cells (Hep2) were used to determine the cytotoxicity of the extracted proteins. The cells were obtained from Nawah, Co. Scientific and maintained in DMEM supplemented with 100 mg/ml streptomycin, 100 units/ml penicillin and 10% heat inactivated fetal bovine serum (FBS). The cells were incubated at 37 °C in humidified CO_2_ incubator (5% v/v). The cytotoxicity and cell viability post exposure to exotoxin A protein extract at 10 and 100 µg/ml were assessed by SRB assay. From a fresh cell culture, 100 µl aliquots of cell suspension (5 × 10^3^) were dispensed in 96 well microtiter plate, supplemented with fresh media and incubated for 24 h. Post incubation, the old media in the plate were discarded and were treated with 100 µl of fresh media containing different concentration of protein solution. The plate was transferred to the incubator for 72 h. then the media was discarded, and the cells were fixed with 150 µl of 10% TCA at 4 °C for 1 h. Afterwards, the cells were washed with distilled water (5 times) then 70 µl of SRB was added (0.4% w/v). The cells were incubated in a dark place at room temperature for 10 min. The dye was discarded, and the cells were washed with 1% acetic acid and air dried overnight. Then, 150 µl of tris buffer was added (10 mM) and the absorbance was measured at 540 nm. The cytotoxic assay was conducted in 3 test replicates against doxorubicin as a positive control and tris buffer as a negative control (Vichai and Kirtikara 2006; Allam et al. 2018).

$${\%\text{ Viability}} = \frac{{\text{Mean OD sample}}}{{\text{Mean OD control}}} \times 100$$

$${\%\text{ Cytotoxicity}} = 100 - \left( {\frac{{\text{Mean OD sample}}}{{\text{Mean OD control}}} \times 100} \right)$$

### Impact of gamma radiation on exotoxin A activity

Impact of low doses gamma radiation on the activity of exotoxin A was examined at 5, 10, 15 and 24 Gy. Overnight growths of *P. aeruginosa* isolate code 22 and 16 in tryptic soy broth (CONDA, Spain) supplemented with 1% glycerol was adjusted at optical density 0.9 and irradiated at dose rate 0.633 rad/s using the Canadian Gamma Cell-40 (137Cs) housed at the National Centre for Radiation Research and Technology (NCRRT), Egyptian Atomic Energy Authority, Cairo, Egypt. Then, the treated cells were subjected to the same extracting procedure, ADP-ribosyl transferase activity and SRB cytotoxicity assays. The impact of gamma radiation on the gene encoding exotoxin A was also depicted via the amplification of the gene from an irradiated culture at 5, 10 and 24 Gy and base deletion was spotted by sequencing analysis.

### Statistical analysis

*P* value for SRB cytotoxicity assay relative to both positive and negative controls was computed using single factor analysis of variance (ANOVA: single factor) and the level of variance was adjusted at 0.05. The analysis was done using Microsoft Excel 2011 Data analysis tool.

## Results

### Identification of the recovered bacterial isolates

A total of 40 isolates formerly recovered from pus samples (wound infection) exhibited green fluorescent pigmentation upon cultivation on Cetrimide agar, from which, four isolates were selected for 16s rRNA identification. *P. aeruginosa* isolate 16 (Accession number: MZ713410.1; CCASU-PA16) and *P. aeruginosa* isolate 22 (Accession number: MZ713405.1; CCASU-PA22) partial 16S ribosomal RNA sequence exhibited 100% identity with *P. aeruginosa* NBRC strain 12689 (NR_113599.1) and ATCC strain 10145 (NR_114471.1) with an average pairwise distance of 0.00993 and 0.00971, respectively. Isolate *P. aeruginosa* isolate 1 partial 16S ribosomal RNA sequence (Accession number: MZ713413.1; CCASU-PA1) scored 99% identity with NBRC strain 12,689 (NR_113599.1) and *P. aeruginosa* DSM strain 50071 (NR_117678.1) with an average pairwise distance 0.0246. Lastly, isolate *P. aeruginosa* isolate 39 partial 16S ribosomal RNA sequence (Accession number: MZ713414.1; CCASU-PA39) recorded 99.81% identity with NBRC strain 12,689 (NR_113599.1) and 97.7% identity DSM strain 50071 (NR_117678.1) with an average pairwise distance 0.02891. The phylogenetic trees and computed pairwise distances for the sequenced 16srRNA genes could be found in Additional file 1: Figs. S1–S4; Tables S1–S4.

### FTIR and UV spectroscopic analysis of lyophilized powder

Additional file 1: Fig. S5 illustrates the FTIR spectroscopy of the lyophilized powder of the expected molecular formula C_8_H_9_N_5_O_2_. A strong broad band appeared at the wave number 2978 to 3654 cm^−1^ suggesting the presence of NH amine salt stretch which is specific for the guanidine group. The appearance of a forked band at 1339 and 1504 cm^−1^, respectively indicated the presence of nitrogen dioxide moiety (NO_2_). The NH bend band appeared at 1580–1617 cm^−1^ whereas the band appeared at 1672 cm^−1^ is attributed to C=N bond. Finally, the C–N for the tertiary amine appeared at 1097–1146 cm^−1^. Combining the bands of the IR spectra all together suggested that formation of 4-nitrobenzylidine aminoguanidine (NBAG). The lyophilized powder was dissolved in 0.1 N HCl and NaOH and the absorbance spectrum was plotted at the UV range 200–600 nm as shown in Additional file 1: Fig. S5. Upon dissolving NBAG in 0.1 N HCl and NaOH, the absorbance maxima appeared at 315 and 372 nm, respectively.

### Mass spectroscopic analysis of lyophilized powder

The Atmospheric pressure chemical ionization mass (APCI-MS) and electrospray ionization mass spectrometers (ESI–MS) of the lyophilized powder denoted a strong base peak at m/z 208.4 with a peak area intensity of 99% as demonstrated by Additional file 1: Fig. S6. This is equivalent to the molecular weight of NBAG in previously published literature.

### ^13^C-NMR spectroscopic analysis of lyophilized powder

The ^13^C-NMR spectrum of lyophilized powder (Additional file 1: Fig. S7) exhibited δ at 148.075 for (C–NO_2_), 139.75, 128.569, and 123.809 for (Aromatic carbons), 155.6 for (C=N) and 144.402 for (C–N). This confirmed the chemical identity of the lyophilized powder, C_8_H_9_N_5_O_2_ and the formation of 4-Nitrobenzylidine aminoguanidine (NBAG).

### Detection of exotoxin A (*tox*A) gene among *P. aeruginosa* strains and sequencing analysis

The product of amplification by polymerase chain reaction appeared as a strong illuminate band at approximately 377 bp. This band appeared in 32 of 40 tested isolates (Additional file 1: Fig. S8). The sequenced amplicon has been submitted into NCBI GenBank database and granted the accession number MZ851972.1. Upon blasting the sequenced fragment of *tox*A with the nucleotide database of NCBI GenBank, it showed 100% identity with *P. aeruginosa* strain ss5 exotoxin A, partial cds (MH373640.1) and strain PA34 exotoxin A gene, partial cds (MW_732029.1). A phylogenetic tree was constructed for the sequenced gene and some selected nucleotide sequences for *toxA* gene sequences retrieved from NCBI and European Nucleotide archive to show the relative relationship between the sequenced gene and previously published literature (Additional file 1: Figs. S9, S10). The pairwise distance was computed and recorded (Additional file 1: Tables S5, S6) and the overall average distance was 0.011. The nucleotide sequence was also blasted against protein database published on uniport, a phylogenetic tree was constructed, and the identity of the translated protein was confirmed (Additional file 1: Fig. S11).

### Changes induced by low doses gamma radiation in exotoxin A gene sequence

Sequencing and amplification of *toxA* gene fragment post exposure to 5, 10 and 24 Gy gamma radiation doses revealed that gamma rays incurred adenine base deletion at the three irradiated doses ((Additional file 1: Fig. S12).

### Determining ADP ribosyl transferase activity of exotoxin A protein extract by UV spectroscopy

**Fig. 1:**
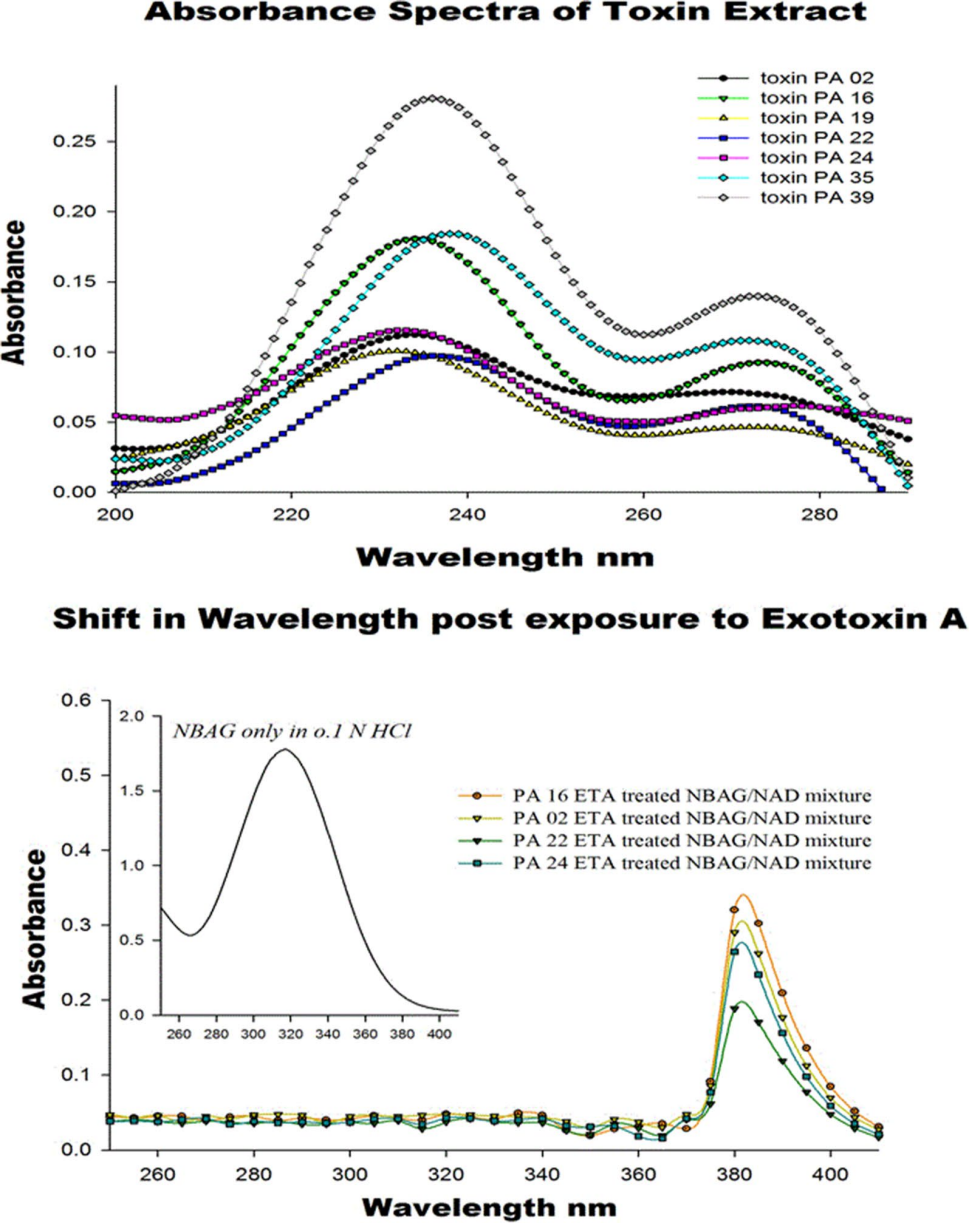
Absorbance spectra of exotoxin A extract alone and the shift in wavelength associated with the addition of exotoxin to NBAG/NAD^+^ mixture

Results showed that, new absorbance maxima aroused post addition of exotoxin A to NBAG and NAD^+^ mixture and incubation at approximately ʎmax ≈ 380–385 nm. These absorbance maxima occurring at ʎmax ≈ 380–385 nm suggested the formation of new product in the reaction tube stimulated by the addition of exotoxin A extract since its ʎmax differ from that of exotoxin A and NBAG. The new ʎmax was similar to the UV spectrum of previously published ADP-ribosylated spectrum of benzylidine-aminoguanidine suggesting that the ADP-ribosyltransferase reaction was initiated by the addition exotoxin A extract. The shift to ʎmax ≈ 380–385 nm after the addition of exotoxin A and absorbance maxima are recorded in Additional file 1: Table S7 (Fig. 1; Additional file 1: Fig. S13). The expected concentration of ADP-ribosylated NBAG formed was computed according to the work conducted by Soman et al. where an 0.1 unit rise in absorbance is equivalent to the formation of 5 × 10–2 µmol/ml ADP ribosylated product. Consequently, the percentage of ADP-ribosylated NBAG formed was calculated.

### Determining ADP ribosyl transferase activity by UV spectroscopy post exposure to gamma radiation

Upon adding the irradiated exotoxin A toxin extract to NBAG and NAD^+^ mixture and measuring the absorbance against a blank reaction tube (as previously described), a prominent reduction in the absorbance at ʎmax ≈ 380–385 nm was recorded (Additional file 1: Fig. S14). The reduction in the absorbance maxima at ʎmax 380–385 nm suggested that the ADP-ribosyltransferase activity of exotoxin A was reduced. Additional file 1: Table S8 demonstrates the expected concentration of ADP-ribosylated NBAG post exposure to gamma irradiated exotoxin A and % reduction in the amount of ADP-ribosylated NBAG formed. The reduction in ADP-ribosylated NBAG produced was computed by subtracting the computed concentration of ADP-ribosylated NBAG post exposure to gamma irradiated exotoxin A from the concentration of ADP-ribosylated post exposure to untreated exotoxin A. Exotoxin A extract obtained from PA isolate 22 did not exhibit a dose dependent reduction in ADP-ribosyltransferase activity. The 24 Gy irradiated exotoxin A extract added to NBAG and NAD^+^ mixture showed the highest ADP-ribosylating activity as depicted by the highest absorbance maxima recorded followed by 15 Gy irradiated exotoxin A whereas the lowest ADP-ribosylating activity was observed in the tube treated with 5 Gy irradiated exotoxin A. On contrast, a dose dependent reduction in ADP-ribosylating activity of PA isolate 16 exotoxin A was observed post exposure to radiation doses 10 and 15 Gy. However, the ADP-ribosylating activity of 24 Gy treated PA 16 exotoxin A was slightly higher than that exhibited by the 10 Gy treated exotoxin A.

### Cytotoxicity of exotoxin A protein extract on cultured *Hep*2 cells

**Fig. 2:**
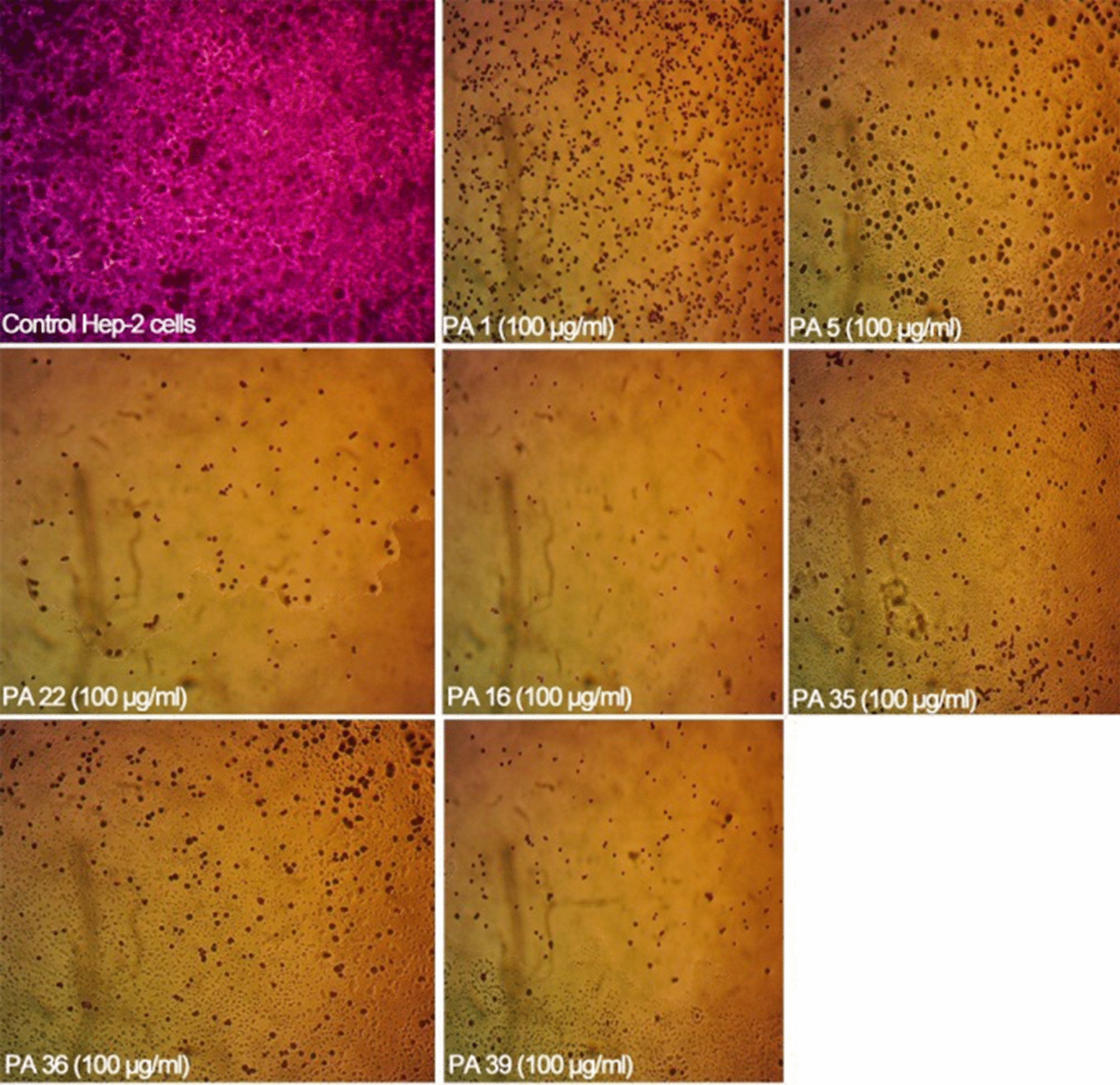
Cytotoxic effects of exotoxin A protein extract at 100 µg/ml on cultured *Hep2* cells versus control untreated *Hep2* cells

The cytotoxicity of exotoxin A protein extract was examined on cultured *Hep2* cells. Quick cytotoxicity screening revealed that at 100 µg/ml, the protein extract exhibited significant cytotoxic effect reducing the cell viability of the cultured cells as shown in Fig. 2. At lower concentration, 10 µg/ml, the cytotoxic effect was nearly abolished, and cells were viable. The values for %cytotoxicity, % cell viability and *P values* are reported in Additional file 1: Table S9. The cytotoxic effects exhibited by exotoxin A protein extracts suggests the ADP-ribosyl transferase activity of exotoxin A intracellular.

### Cytotoxicity of exotoxin A protein extract on cultured Hep-2 cells post exposure to gamma radiation

**Fig. 3:**
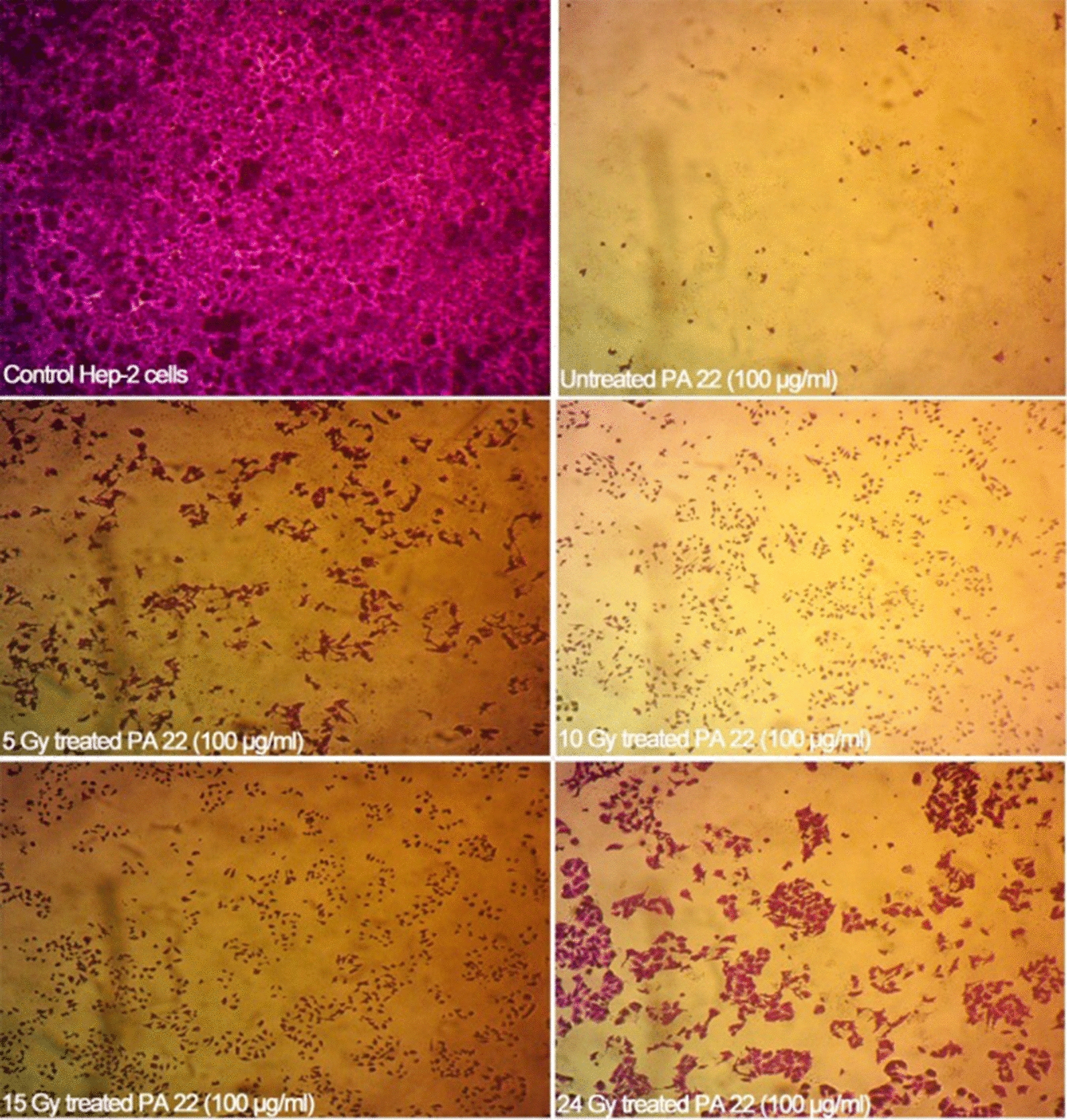
Cytotoxic effect of gamma irradiated exotoxin A protein extract at 100 µg/ml on cultured *Hep2* cells versus control untreated *Hep2* cells

**Fig. 4:**
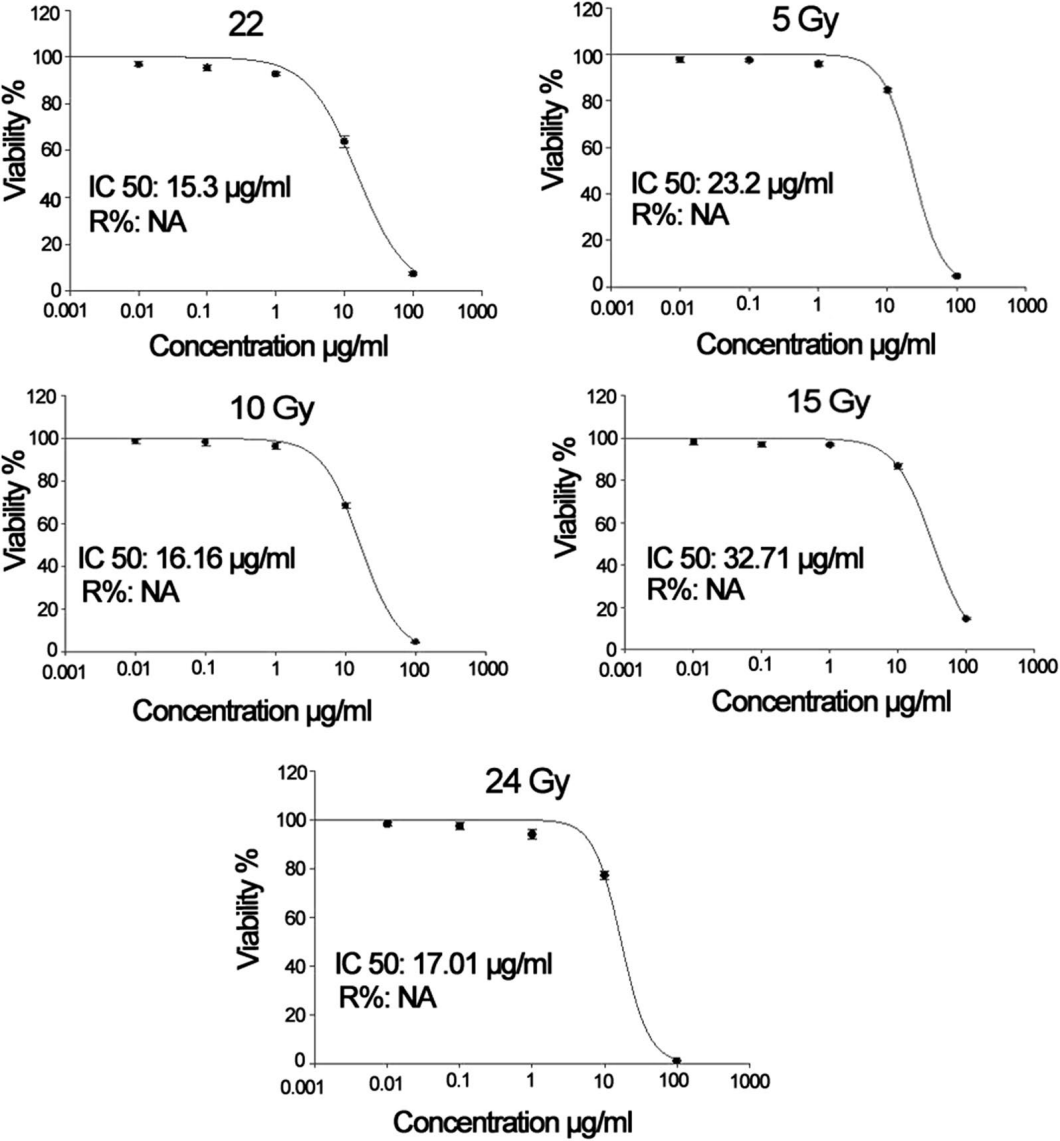
Plot of percentage viability against the concentration of protein extracts (µg/ml) prior and post exposure to low doses gamma radiation and IC50 values

Irradiated exotoxin A extracts of PA 22 isolate exhibited cytotoxic effects on cultured Hep-2 cells at 100 µg/ml as demonstrated in Fig. 3. However, the IC50 µg/ml was increased when compared to the untreated exotoxin A effect suggesting that the cytotoxicity was reduced by gamma radiation (Additional file 1: Table S10, Fig. 4). The lowest increase in the cell viability and highest cytotoxic effect post exposure to gamma irradiated protein extract at 100 µg/ml was noted with 15 Gy gamma irradiated extract that showed a 6.65% increase in cellular viability. At 5 Gy irradiated protein extract at 10 µg/ml exhibited the lowest cytotoxic effect that was associated with a 22.74% increase in cellular viability. The values of %cell viability, %increase in the cell viability post exposure to gamma irradiated protein and p values are stated in Additional file 1: Table S10.

### Determination of ADP ribosyl transferase activity using HPLC

**Fig. 5:**
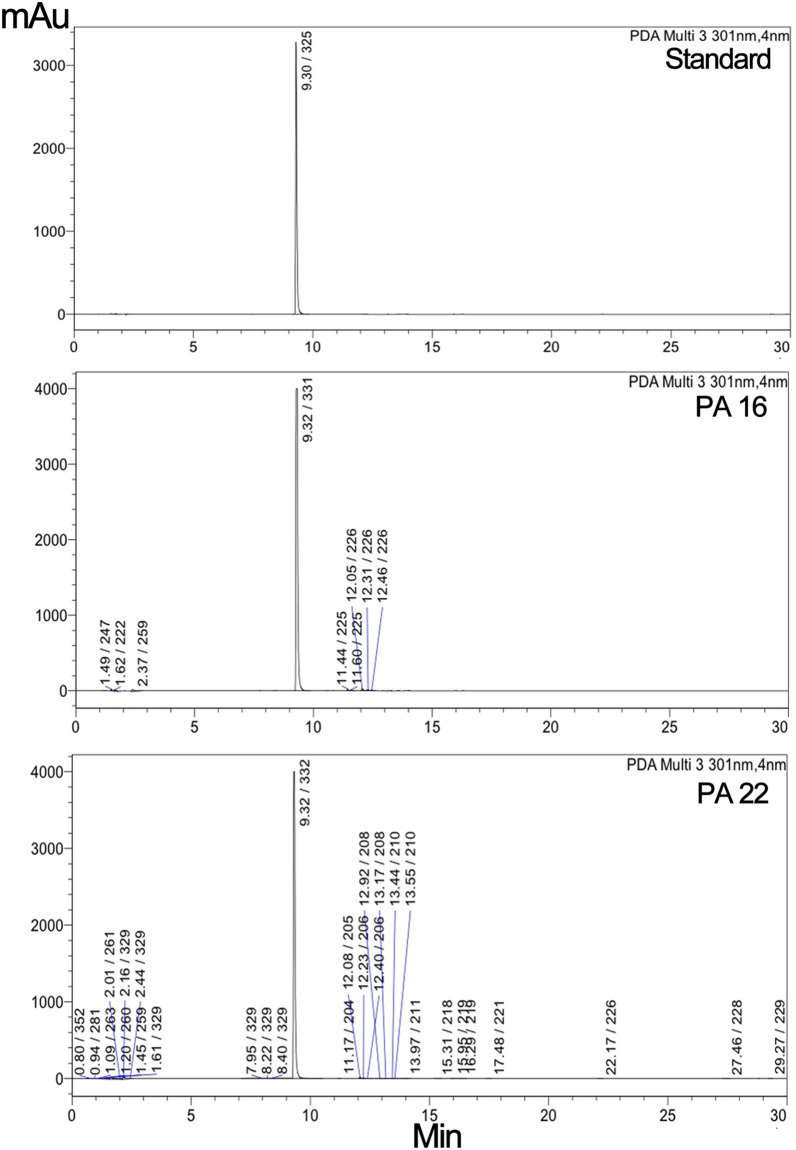
HPLC Chromatogram for standard and exotoxin A protein extract treated NBAG reactions at 301 nm

**Fig. 6:**
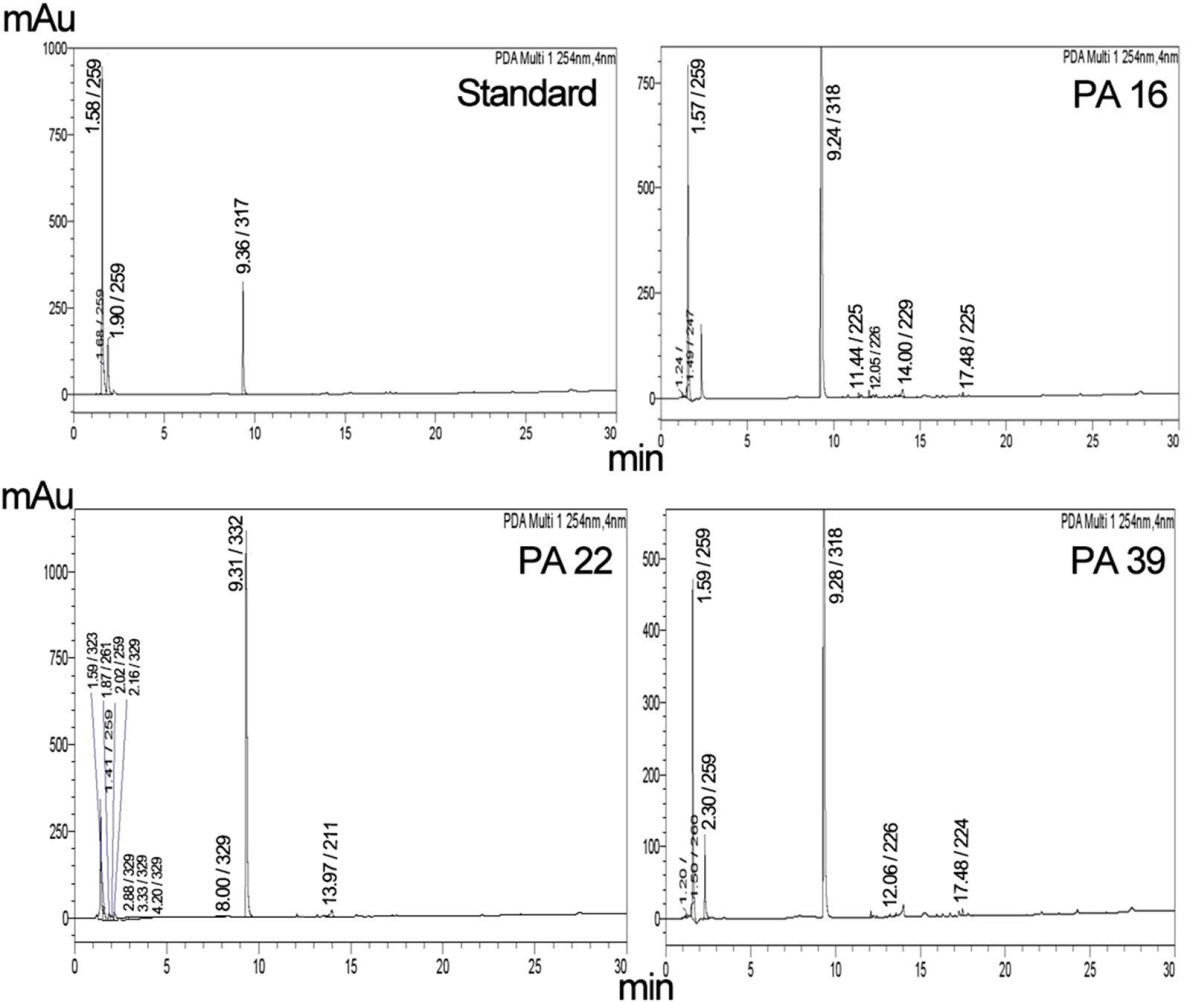
HPLC Chromatogram for standard and exotoxin A protein extract treated NBAG reactions at 254 nm

**Fig. 7:**
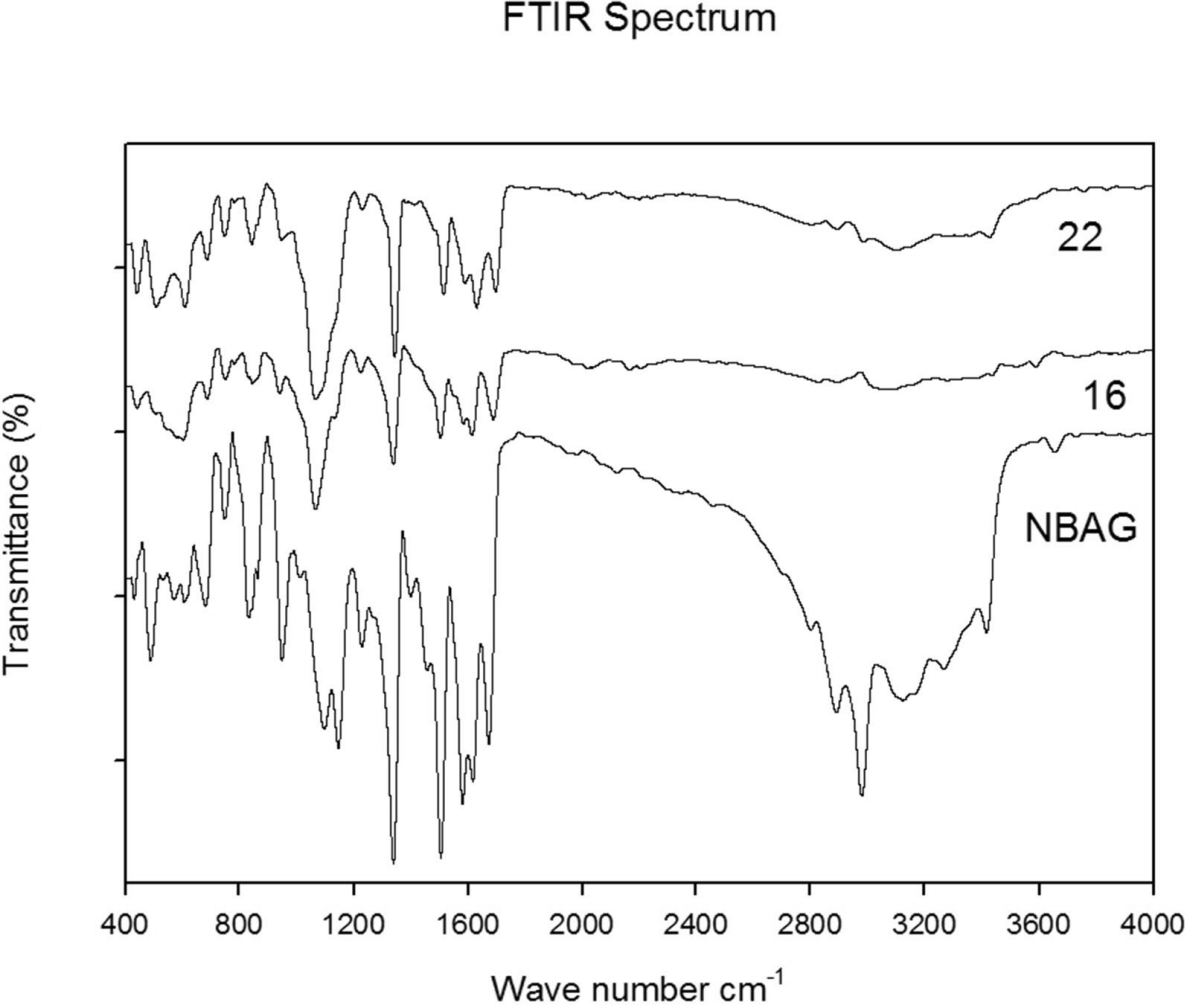
FTIR spectrum for the precipitate formed post exposure of NBAG and NAD^+^ to exotoxin A protein Extract

HPLC was used to elute the ADP-ribosylated product from the treated NBAG and NAD^+^ mixture. The rise of minor peaks around the retention time 11–14 min among the exotoxin A treated NBAG and NAD^+^ mixtures indicated that ADP-ribosyltransferase reaction took place (Figs. 5, 6; Additional file 1: Figs. S15–S19). Incubating the NBAG and NAD^+^ together in the standard tube didn’t exhibit these new minor peaks indicating that a reaction took place after the addition of exotoxin A extract into the reaction tubes. Further, the peak splitting around the retention time 1.4–3 min which is also absent in NAD^+^ and NBAG^+^ only mixtures suggested the breakage of the C–N bond in NAD^+^ by the addition of exotoxin A extract (Figs. 5, 6). Moreover, the ʎmax of the minor peaks is slightly different from the ʎmax of the major NBAG peak which indicated that the minor peaks produced acquire structural similarities to the parent compound (Table 1). FTIR spectrum of the retrieved precipitate from NBAG and NAD^+^ mixtures treated by exotoxin A revealed the loss of the NH amine stretch band and the appearance of a band at 1100 cm^−1^ that indicates the presence of phenolic OH bend associated with the forked band of NO_2_ at approximately 1339 and 1504 cm^−1^ suggested the attachment of ADP ribose moiety to nitrobenzene (Fig. 7). Table 1 shows the amount of ADP ribosylated product eluted by HPLC after adding exotoxin A to NBAG and NAD^+^ mixture.

**Table 1:** Concentration of ADP ribosylated NBAG and percent ADP ribosylated NBAG formed post exposure to exotoxin A protein extract at 301 nm

*P. aeruginosa* strains (Code number of tested isolate)	Retention time at 301 nm	ʎ max of the minor peaks	Peak area	ADP-ribosylated NBAG formed in M	Percent ADP ribosylated NBAG formed
PA 1 NBAG peak	9.33	329/227	18,583,078	0.01	
PA 1 new peaks	12.16	228/340	45,548	1.46 × 10^–5^	0.31
	12.29	229/327	53,355	1.71 × 10^–5^	
PA 5 NBAG peak	9.47	304/325/230	30,528,050	0.01	
PA 5 new peaks	12.29	225/327	35,634	1.16 × 10^–5^	0.23
	13.58	226/268	33,613	1.101 × 10^–5^	
PA 16 NBAG peak	9.32	331/228	21,872,503	0.01	
PA 16 new peaks	11.44	225/327	77,762	3.55 × 10^–5^	1.71
	11.6	225/327	60,768	2.78 × 10^–5^	
	12.05	226/339	123,413	5.64 × 10^–5^	
	12.31	226/327	63,771	2.91 × 10^–5^	
	12.46	226/327	49,347	2.26 × 10^–5^	
	11.44	225/327	77,762	3.55 × 10^–5^	
	11.6	225/327	60,768	2.77 × 10^–5^	
	12.05	226/339	123,413	5.64 × 10^–5^	
PA 22 NBAG peak	9.32	332	18,955,192	0.01	
PA 22 new peaks	11.17	204/266/329/367/288	3050	1.61 × 10^–6^	0.85
	12.08	205/340	62,448	3.29 × 10^–5^	
	12.23	206/329	22,983	1.212 × 10^–5^	
	12.4	206/265/329/368	3215	1.7 × 10^–6^	
	12.92	208/265/329/369	2151	1.13 × 10^–6^	
	13.17	208/323/369	6124	3.23 × 10^–6^	
PA 35 NBAG peak	9.43	334/231	31,719,819	0.01	
PA 35 new peaks	12.16	224/339	44,585	1.41 × 10^–5^	0.42
	12.29	225/327	48,434	1.53 × 10^–5^	
	13.58	225/268	42,470	1.34 × 10^–5^	
PA 39 NBAG peak	9.33	329/227	18,583,078	0.01	
PA 39 new peaks	12.08	226/340	62,029	3.34 × 10^–5^	0.33

Conc. = concentration; Concentration of ADP ribosylated NBAG = (area unknown/area known) × concentration of known (NBAG); Percent reduction in NBAG conc. = (total concentration of ADP ribosylated NBAG/concentration of NBAG) × 100

### Impact of low doses of gamma radiation on ADP-ribosyltransferase activity of exotoxin A by HPLC

**Fig. 8:**
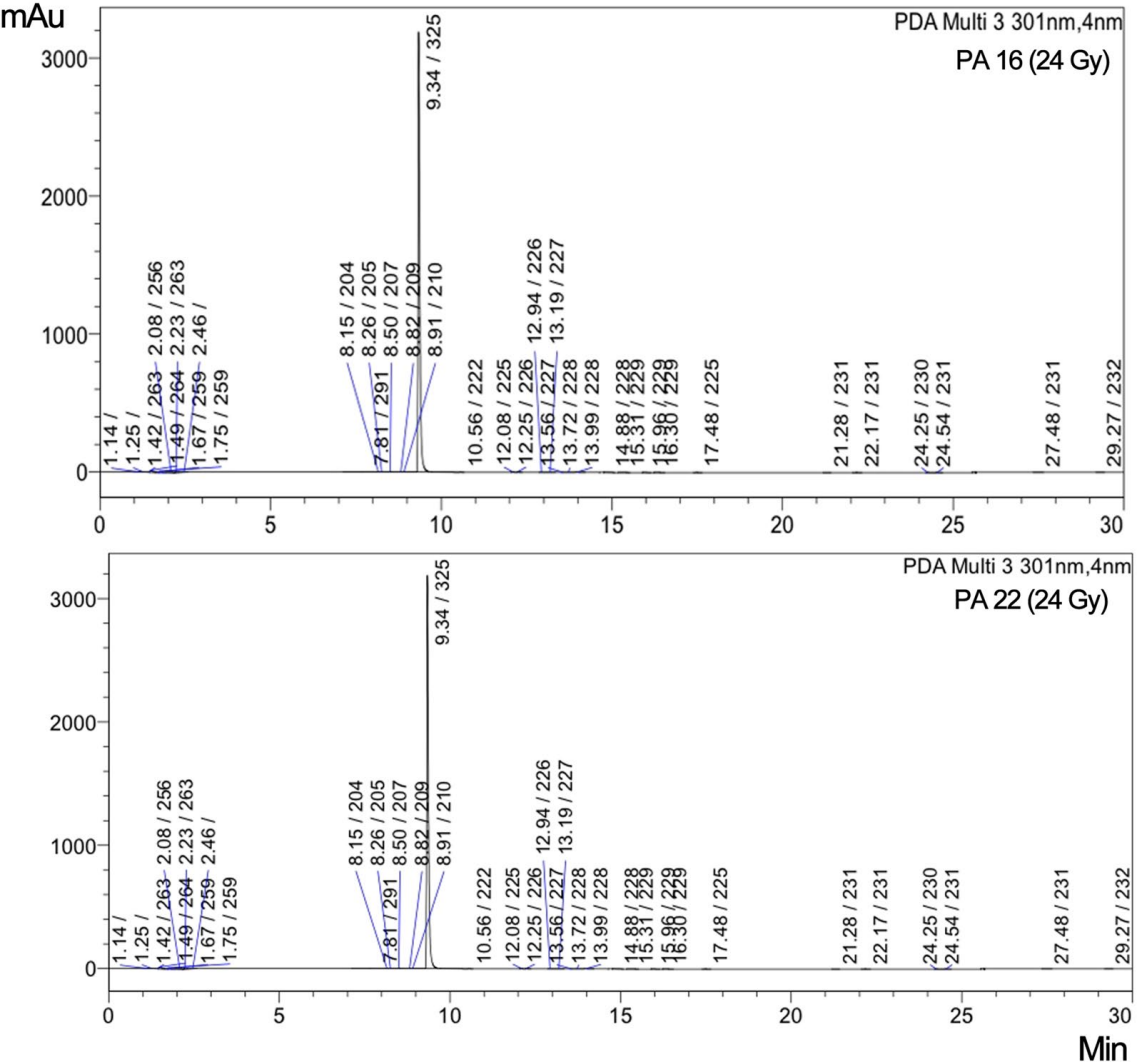
HPLC Chromatogram for standard and irradiated exotoxin A protein extract treated NBAG reactions at 301 nm

Gamma irradiated exotoxin A exerted lower ADP-ribosylating activity as noted by the disappearance of the minor peaks in HPLC chromatograms (Additional file 1: Figs. S20–S23). It was noted that the HPLC chromatogram of 5, 10 and 15 Gy irradiated PA 22 exotoxin A treated NBAG and NAD^+^ mixture didn’t differ from the NBAG and NAD^+^ mixture only chromatogram suggesting that ADP-ribosylating reaction didn’t take place. However, the 24 Gy irradiated PA 22 exotoxin A exerted an ADP-ribosylating effect on NBAG and NAD^+^ mixtures yet lower than that exerted by the untreated PA 22 exotoxin A. In similar manner, the chromatogram of 10 and 15 Gy irradiated PA 16 exotoxin A treated NBAG and NAD^+^ mixture suggested the absence of the ADP-ribosylation reaction as noted by similarity with NBAG and NAD^+^ mixture only chromatogram. Whereas the 24 Gy irradiated PA 16 exotoxin A exhibited an ADP-ribosylating effect on the tested mixture that is lower than the untreated PA 16 exotoxin. Table 2 and Fig. 8 shows the amount of ADP-ribosylated product formed.

**Table 2:** Concentration of ADP ribosylated NBAG formed post exposure to irradiated exotoxin A protein extract and percent reduction compared to the un-irradiated exotoxin A

Radiation dosage	Retention time at 301 nm	Peak area	ʎ max	Conc. of unknown (ADP-ribosylated products)	Percent of ADP-ribosylated products formed	Percent reduction in the amount of ADP-ribosylated product formed
*P. aeruginosa* 16 (24 Gy) NBAG peak	9.38	11,632,146	314/228	0.01		
*P. aeruginosa* 16 (24 Gy) new peaks	10.6	1300	223/291/327	1.118 × 10^–6^	0.73	0.98
	12.14	11,518	225/340	9.902 × 10^–6^		
	12.24	17,167	225/327	1.476 × 10^–6^		
	13.17	2415	225	2.076 × 10^–6^		
	13.54	8416	226	7.235 × 10^–6^		
*P. aeruginosa* 22 (24 Gy) NBAG peak	9.34	10,739,513	325/225	0.01		
*P. aeruginosa* 22 (24 Gy) new peaks	10.56	2654	222/291/269/327	2.5 × 10^–6^	0.71	0.14
	12.08	21,741	225/340	2.5 × 10^–6^		
	12.25	16,007	226/331	1.49 × 10^–5^		
	12.94	1606	226/331	1.5 × 10^–6^		
	13.19	2474	227/327	2.3 × 10^–6^		
	13.56	5403	227	5 × 10^–6^		
	13.72	4247	228/327	4 × 10^–6^		

Conc. of ADP ribosylated NBAG = (area unknown/area known) × conc. of NBAG; Percent ADP ribosylated NBAG formed = (total conc. of ADP ribosylated NBAG/conc. of NBAG) × 100; Percent reduction compared to un-irradiated strain = percent of ADP ribosylated NBAG formed for un-irradiated – percent of ADP ribosylated NBAG formed for irradiated

## Discussion

This work investigates the ADP ribosyl transferease activity of exotoxin A extracted from *P. aeruginosa* isolates and the impact of low doses gamma radiation on it. Determining the ADP- ribosyl transferase activity of the exotoxin A foretells a future use for this toxin in anticancer therapy and the manufacture of recombinant immunotoxin. First, from the 40 recovered isolates, *toxA* gene was found in 32 isolates (80%). The identity of the *toxA gene* isolated and amplified from the clinical isolates was confirmed upon blasting the nucleotide sequence with exotoxin A nucleotide sequences published on NCBI GenBank and European Nucleotide Archives (ENA). Following the genotypic detection of *toxA* gene among the retrieved isolates, the toxin was extracted from *P. aeruginosa* isolates in its stage II form as previously described by Liu et al (1973). The zinc acetate was added to the culture supernatant to concentrate the exotoxin A secreted by *P. aeruginosa* isolates and the stage II toxin is the toxin that has been salted out from the dialysed solution by ammonium sulphate. After the salting out, the ADP-ribosyl transferase activity was explored. Interestingly, the ADP ribosyl transferase activity of exotoxin A differs from that of exoenzyme S that it can be detected in in vitro assays. Unlike exoenzyme S, exotoxin A doesn’t require eukaryotic protein (Factor Activating exoenzyme S), to exhibit its enzymatic activity (Coburn et al. 1991). Moreover, exoenzyme S catalyzes the transfer of ADP-ribose portion of NAD to numerous proteins in crude wheat germ extracts only; it cannot utilize the transfer of ADP ribose moiety to nucleic acids or other chemical compounds (Aktories 2012).

In this work, 4-nitrobenzylidine aminoguanidine (NBAG) was synthesized to be the acceptor molecule for the ADP-ribosyltransferease activity of exotoxin A. The synthesis technique was successful as proven by FTIR, UV, ESI, APCI and C^13^-NMR spectrums as revealed in our results. Both ʎ_max_ of NBAG in 0.1 N HCl and 0.1 N NaOH was identical to the original compound synthesized by Soman et al. (1983). The NH amine salt stretch of the FTIR spectrum is a characteristic feature for the guanidine group of NBAG and C=N band at 1672 cm^−1^ denotes the attachment of the nitrobenzene moiety with aminoguanidine group. Further, the mass and ^13^C-NMR spectroscopic analysis confirmed the molecular identity of the compound. The strong peak of mass spectrum appeared at m/z 208 is equivalent to molecular weight of NBAG whereas the peaks abundant at 155.6 and 144.4 in ^13^C-NMR spectrum denotes the existence of C=N and C–N bonds (Wu et al. 2019). Soman et al. developed the applied analytical method in this study that harness non-radioactive nicotinamide adenine dinucleotide (NAD^+^) and guanylhydrazones of *p*-nitrobenzaldehyde in depicting ADP-ribosyltransferase activity of Cholera toxin. This method is reproducible and obtainable since it incorporates no isotopes and the synthesis of guanylhydrazones of *p*-nitrobenzaldehyde can be easily achieved. Guanylhydrazones of *p*-nitrobenzaldehyde accept the ADP ribose group form ADP-ribosyltransferases resulting in color changes that facilitate the observation of the reaction by spectrophotometric assays (Soman et al. 1983).

The reaction tube contained dithioerithritol (DTE) that was pre-incubated with the exotoxin A protein extract at 35 °C for 30 min prior to the addition of NAD^+^ and NBAG. This is crucial step to initiate the enzymatic activity of exotoxin A in the protein extract. It’s previously reported that the exotoxin A is produced as a proenzyme that lacks its enzymatic activity. The toxin exhibits its activity either by proteolytic cleavage or denaturation and reduction by Urea or dithioerithritol (DTE) (Iglewski et al. 1978; Wick et al. 1990). After the pre-incubation period, the exotoxin A extract was added to the NAD^+^ and NBAG mixture and a new ʎmax at ≈380 nm aroused. The new peak ʎmax differed from the ʎmax of NBAG and the exotoxin A alone suggesting the formation of a new compound in the reaction tube. It was also noted that the new pronounced ʎmax at ≈380–385 nm was similar to the ʎmax of the ADP-ribosylated gyanyl hydrazone DEA-BAG indicating that the ADP-ribosyltransferase reaction took place after the addition of the exotoxin A to reaction tube (Narayanan et al. 1989). Moreover, the shift associated with the new ʎmax suggested the deprotonation of the guanidine group in NBAG (Zhou et al. 2013) which further support the attachment of ADP-ribose moiety and that the ADP-ribosyltransferase reaction took place. Soman et al. (1983) reported that a slight change in the absorbance spectrum of NBAG by 0.1 units is equivalent to the formation of 5 µmol/ml of ADP ribosylated product under the stated reaction condition. The low concentration of ADP-ribosylated product formed post exposure to exotoxin A protein extract is attributed to the presence of the NO_2_ at the Para-position of the NBAG. The NO_2_ group is a strong electron withdrawing group that generates an electron deficient condition at the reaction site (guanidine group) which in turns lowers the kinetic efficiency (Michaelis constant K_m_ and catalytic rate K_Cat_) of the ADP-ribosylation reaction mediated by exotoxin A extract. Soman et al. (1986) manifested that the presence of electron donating groups at the Para position of the (benzy1idineamino) guanidine such as C_6_H_5_, OC(CH_3_)_3_, OCH_3_, OC_2_H_5_, N(CH_3_)_2_ and N(C_2_H_5_)_2_ is associated with higher Michaelis constant (K_m_) as they donate electron to the reaction site i.e. guanidine group facilitating the turnover of substrate to product by the ADP-ribosyltransferase enzymes (enhance the K_cat_). The small amount of ADP-ribosylated NBAG detected by UV spectrophotometry was also equivalent with previously published reports of Soman et al. (1991) whom retrieved the ADP-ribosyltransferase activity from different T cell hybridoma cell lines using NBAG by a rate 0.035, 0.047, and 0.1 µmol/h/mg of protein.

In the HPLC chromatogram, it was noted that multiple peaks aroused around the retention time 2.88 to 4.2 in the chromatogram of reaction treated with strains *Pa* 22 and *Pa* 16 exotoxin A protein extracts. The appearance of these peaks indicates the release of ADP-ribose from NAD^+^ confirming the ADP-ribosyl transferase activity of exotoxin A protein extract (Tak et al. 2019). Soman et al. (1983) also reported that ADP-ribosyltransferases could hydrolyze NAD^+^ to ADP ribose sugar and Nicotinamide. The appearance of peaks at the retention time 12 to 14 min suggested the ADP-ribosylation of NBAG. The ADP-ribosylated products for the guanylhydrazones derivatives appear as discrete peaks after the major peak of NBAG (Soman et al. 1984; Peterson 1990; Donnelly et al. 1996). This is evidenced by the absence of these minor peaks in the HPLC chromatogram of the NBAG/NAD^+^ only mixture and the similarity of the ʎmax of the new peaks to the ADP-ribosylated products of (benzy1idineamino) guanidine (Soman et al. 1983). The concentration of ADP-ribosylated eluted by HPLC is relatively equivalent to the amount detected by UV spectroscopy which can be explained by the presence of NO_2_ group at the Para position of NBAG (Soman et al. 1986). Moreover, FTIR spectra of the reaction precipitate denoted the absence of the NH amine stretch which evidenced the acceptance of the ADP-ribosyl moiety by the NBAG and that the ADP-ribosyl transferase reaction took place.

The cytotoxicity of exotoxin A protein extract was tested on head and neck carcinoma cells (*Hep2*) and it was noted that 100 µg/ml was sufficient to induce cellular death of the cultured cells. This observation suggested that the toxin exerted its activity intracellular and ADP ribosylated EF-2 blocking the protein synthesis and inducing cellular apoptosis. The cytotoxicity of the protein extract is coherent with previously published works that indicated the usage of *P. aeruginosa* exotoxin A in anticancer therapy. Wang et al. composed a recombinant immunotoxin from the enzymatic moiety of ExoA, domain III, and HN3 antibody that targets Glypican-3 (GPC3) antigens that are overexpressed in hepatocellular carcinoma (HCC). The study indicated that PE 40, domain III of exotoxin A, acquires a strong cytotoxic effect on cultured hepatocellular carcinoma cells (Wang et al. 2017). The choice of low doses gamma radiation was based on the fact that the toxin has been coupled with radiotherapy in treatment of several cancers. Radiotherapy applies low fractionated doses to overcome the acute toxicity associated with large single fractions of radiations that allows cellular repair, redistribution, reoxygenation and repopulation post exposure (Mitchell 2013). The impact of low doses of radiation on the microbial toxin activity in in vivo and in vitro models hasn’t been clearly identified yet. Interestingly, exposure to gamma radiation reduced the absorbance maxima and percentage of ADP ribosylated products formed in HPLC reaction indicating that exposure to low doses gamma radiation lowered the ADP ribosyltransferase activity.

Additionally, the cytotoxicity of the irradiated proteins was lowered, and the cells seemed to acquire resistance against the toxin. This effect might be attributed to the oxidative modification incurred by ionizing radiation in the cellular proteins that disrupts their functions. These oxidative modifications include direct amino-acid oxidation, oxidative cleavage of the protein backbone and carbonylation of proteins. Oxidative changes are usually associated with conformational changes that alter the enzymatic activity (Reisz et al. 2014). Such effect was previously reported with catalase and protein tyrosine phosphatases enzymes where a mild signal of ionizing radiation associated with the formation of reactive oxygen species stimulates the nitrosylation of these enzymes and reducing their activity (Barrett et al. 2005). It was also noted that radiation induced base deletion in *toxA* gene. At the three tested radiation doses, 5, 10 and 24 Gy, adenine base was deleted. This could be explained by the induction of abasic sites post exposure to gamma radiation (Sudprasert et al. 2006; Cadet et al. 1999) due to the generation of reactive oxygen species. The loss of Adenine consequently alters the amino acid sequence in the protein extract which in turn affects the enzymatic activity of Exotoxin A protein extract. The absence of dose dependent manner could be explained by the constant dose rate that has been applied, as the rate of radiation damage is highly dependent on the energy level rather than the radiation dose value (Min et al. 2003).

## Conclusion

Exotoxin A gene was prevalent in 80% of the recovered clinical isolates. Synthesis of 4-nitrobenzylidene aminoguanidine (NBAG) was successful as proven by FTIR, UV, Mass and ^13^C-NMR spectra. Exotoxin A protein extract exhibited an ADP-ribosyltransferase activity as determined by the changes in the NBAG absorbance spectra post exposure to toxin (Shift to ʎmax ≈380–385 nm), fragmentation of NAD^+^ peaks and rise of new peaks after the major NBAG peak in in HPLC chromatograms which indicates the formation of ADP-ribosylated product. Exotoxin A protein extract induced prominent cytotoxic effect on cultured *Hep-2* cells that suggests the activity of the enzyme intracellularly. Exposure to low doses gamma radiation at 5, 10 and 15 Gy reduced the ADP-ribosyl transferase activity of the exotoxin A which is denoted by the reduction in ADP-ribosylated NBAG formed determined by UV spectroscopy and it’s absence in elute of HPLC chromatogram. The 24 Gy irradiated exotoxin A exerted an ADP-ribosylating effect yet lower than that exerted by the untreated toxin extract. The changes associated with gamma radiation treatment might be alerting upon coupling radiotherapy with anticancer therapy including *P. aeruginosa* exotoxin A.

## Supplementary Information

**Additional file 1.** **Figure S1** Bootstrap tree for *P. aeruginosa* isolate (22) 16S ribosomal RNA sequence against 16S ribosomal RNA sequences retrieved from NCBI GenBank. **Figure S2** Bootstrap tree for *P. aeruginosa* isolate (16) 16S ribosomal RNA sequence against 16S ribosomal RNA sequences retrieved from NCBI GenBank. **Figure S3** Bootstrap tree for *P. aeruginosa* isolate (1) 16S ribosomal RNA sequence against 16S ribosomal RNA sequences retrieved from NCBI GenBank. **Figure S4** Bootstrap tree for *P. aeruginosa* isolate (39) 16S ribosomal RNA sequence against 16S ribosomal RNA sequences retrieved from NCBI GenBank. **Figure S5** UV and FTIR absorbance spectra for lyophilized powder nitrobenzylidine aminoguanidine (NBAG). **Figure S6** Mass spectra for lyophilized powder, nitrobenzylidine aminoguanidine (NBAG). **Figure S7** ^13^C-NMR spectrum for lyophilized powder, nitrobenzylidine aminoguanidine (NBAG). **Figure S8** Agarose Gel electrophoresis for the detection of *tox*A amplicons. **Figure S9** Bootstrap tree for partial sequence of *tox*A gene (Exotoxin A Partial *P. aeruginosa* isolate 1) blasted against *tox*A gene records retrieved from NCBI GenBank. **Figure S10** Bootstrap tree for partial sequence of *tox*A gene (Exotoxin A Partial *P. aeruginosa* isolate 1) blasted against *tox*A gene records retrieved from European nucleotide Archives (ENA). **Figure S11** Bootstrap tree for translated *tox*A gene (Exotoxin A (Fragment) OS *P. aeruginosa* isolate 1) blasted against exotoxin A protein records retrieved from Uniprot Database. **Figure S12** Alignment of *tox*A sequence and deletion in the Adenine base. **Figure S13** The absorbance spectra of ADP ribosylated nitrobenzylidine aminoguanidine (NBAG). **Figure S14** Reduction in the absorbance maxima post exposure to gamma irradiated exotoxin A protein extract. **Figure S15** HPLC chromatogram of *P. aeruginosa* isolate 1 at 301 nm. **Figure S16** HPLC chromatogram of *P. aeruginosa* isolate 5 at 301 nm. **Figure S17** HPLC chromatogram of *P. aeruginosa* isolate 35 at 301 nm. **Figure S18** HPLC chromatogram of PA 39 isolate at 301 nm. **Figure S19** HPLC chromatogram of *P. aeruginosa* isolate 22 irradiated at 5Gy at 301 nm. **Figure S20** HPLC chromatogram of *P. aeruginosa* isolate 22 irradiated at 15Gy at 301 nm. **Figure S21** HPLC chromatogram of *P. aeruginosa* isolate 22 irradiated at 10Gy at 301 nm. **Figure S22** HPLC chromatogram of *P. aeruginosa* isolate 16 irradiated at 15Gy at 301 nm. **Figure S23** HPLC chromatogram of *P. aeruginosa* isolate 16 irradiated at 10Gy at 301 nm. **Table S1** Computed Pairwise distance between *P. aeruginosa* isolate 22, 16S ribosomal RNA and 16S ribosomal RNA sequences retrieved from NCBI GenBank. **Table S2** Computed Pairwise distance between *P. aeruginosa* isolate 16, 16S ribosomal RNA and 16S ribosomal RNA sequences retrieved from NCBI GenBank. **Table S3** Computed Pairwise distance between *P. aeruginosa* isolate 1, 16S ribosomal RNA and 16S ribosomal RNA sequences retrieved from NCBI GenBank. **Table S4** Computed Pairwise distance between *P. aeruginosa* isolate 39, 16S ribosomal RNA and 16S ribosomal RNA sequences retrieved from NCBI GenBank. **Table S5** Computed Pairwise distance between Exotoxin A partial sequence *P. aeruginosa* isolate 1 and *tox*A gene sequences retrieved from NCBI GenBank. **Table S6** Computed Pairwise distance between Exotoxin A partial sequence *P. aeruginosa* isolate 1 and *tox*A gene sequences retrieved from European nucleotide archive (ENA) and NCBI GenBank. **Table S7** Absorbance maxima and expected amount of ADP-ribosylated NBAG formed by *P. aeruginosa* exotoxin A protein extract. **Table S8** Absorbance maxima post exposure to gamma irradiated exotoxin A extract and percent reduction in ADP-ribosylated NBAG formed. **Table S9** Percent cytotoxicity and viability for exotoxin A protein extract on cultured *Hep2* cells. **Table S10** Percent cell viability and increase in cellular viability post exposure to irradiated *P. aeruginosa* exotoxin A protein extract.

## Data Availability

All data generated or analyzed during this study are included in this published article.

## Abbreviations

NAD^+^: Nicotinamide adenine dinucleotide
NBAG: 4-Nitrobenzylidine aminoguanidine
ADP-ribose: Adensoine diphosphate ribose
HPLC: High performance liquid chromatography
UV spectroscopy: Ultraviolet visible spectroscopy
FTIR: Fourier-transform infrared spectroscopy
ESI: Electron Spray Ionization mass spectroscopy
APCI: Atmospheric pressure chemical ionization mass spectroscopy
^13^C-NMR: Carbon 13 nuclear magnetic resonance
Gy: Gray
*Hep-2*: Head and neck carcinoma cells
*toxA*: Exotoxin A gene
