# Supplementary material for: ADP-ribosyl transferase activity and gamma radiation cytotoxicity of Pseudomonas aeruginosa exotoxin A
Source: AMB Express. 2021 Dec 22;11:173. doi: 10.1186/s13568-021-01332-3 (PMC8695647; doi:10.1186/s13568-021-01332-3)
Supplement: Supplementary file 1 — Additional file 1. Figure S1 Bootstrap tree for P. aeruginosa isolate (22) 16S ribosomal RNA sequence against 16S ribosomal RNA sequences retrieved from NCBI GenBank. Figure S2 Bootstrap tree for P. aeruginosa isolate (16) 16S ribosomal RNA sequence against 16S ribosomal RNA sequences retrieved from NCBI GenBank. Figure S3 Bootstrap tree for P. aeruginosa isolate (1) 16S ribosomal RNA sequence against 16S ribosomal RNA sequences retrieved from NCBI GenBank. Figure S4 Bootstrap tree for P. aeruginosa isolate (39) 16S ribosomal RNA sequence against 16S ribosomal RNA sequences retrieved from NCBI GenBank. Figure S5 UV and FTIR absorbance spectra for lyophilized powder nitrobenzylidine aminoguanidine (NBAG). Figure S6 Mass spectra for lyophilized powder, nitrobenzylidine aminoguanidine (NBAG). Figure S7 13C-NMR spectrum for lyophilized powder, nitrobenzylidine aminoguanidine (NBAG). Figure S8 Agarose Gel electrophoresis for the detection of toxA amplicons. Figure S9 Bootstrap tree for partial sequence of toxA gene (Exotoxin A Partial P. aeruginosa isolate 1) blasted against toxA gene records retrieved from NCBI GenBank. Figure S10 Bootstrap tree for partial sequence of toxA gene (Exotoxin A Partial P. aeruginosa isolate 1) blasted against toxA gene records retrieved from European nucleotide Archives (ENA). Figure S11 Bootstrap tree for translated toxA gene (Exotoxin A (Fragment) OS P. aeruginosa isolate 1) blasted against exotoxin A protein records retrieved from Uniprot Database. Figure S12 Alignment of toxA sequence and deletion in the Adenine base. Figure S13 The absorbance spectra of ADP ribosylated nitrobenzylidine aminoguanidine (NBAG). Figure S14 Reduction in the absorbance maxima post exposure to gamma irradiated exotoxin A protein extract. Figure S15 HPLC chromatogram of P. aeruginosa isolate 1 at 301 nm. Figure S16 HPLC chromatogram of P. aeruginosa isolate 5 at 301 nm. Figure S17 HPLC chromatogram of P. aeruginosa isolate 35 at 301 nm. Figure S18 HPLC chromat [file 13568_2021_1332_MOESM1_ESM.pdf]

-Journal name: AMB Express

-Manuscript Title: ADP-ribosyl transferase activity and gamma radiation cytotoxicity of *Pseudomonas aeruginosa* Exotoxin A

Radwa N. Morgan[1], Sarra E. Saleh[2], Khaled M. Aboshanab[2*], Hala A. Farrag[1]

[1]National Centre for Radiation Research and Technology (NCRRT), Drug Radiation Research Department, Egyptian Atomic Energy Authority (EAEA), Ahmed El-Zomor Street, Nasr city, Cairo, 11787, Egypt

[2] Microbiology and Immunology Department, Faculty of Pharmacy, Ain Shams University, African union organization Street, Abbassia, Cairo, 11566, Egypt

Correspondence: Khaled M. Aboshanab (PhD)**,** Professor of Microbiology and Immunology and Vice Dean of Graduate studies, Faculty of Pharmacy, Ain Shams University**,** Cairo, Egypt.

E-mail: aboshanab2012@pharma.asu.edu.eg,

Tel: (202)28429040

Mobile: (002)01007582620

Fax: (202)24051107

https://orchid.org/0000-0002-7608-850X

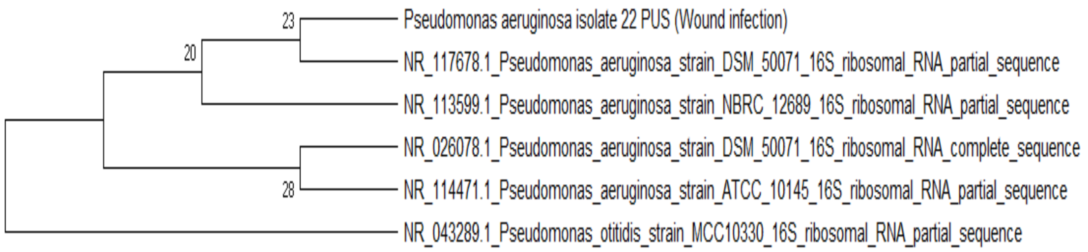

Fig. S1 Bootstrap tree for *P. aeruginosa* isolate (22) 16S ribosomal RNA sequence against 16S ribosomal RNA sequences retrieved from NCBI GenBank**.**

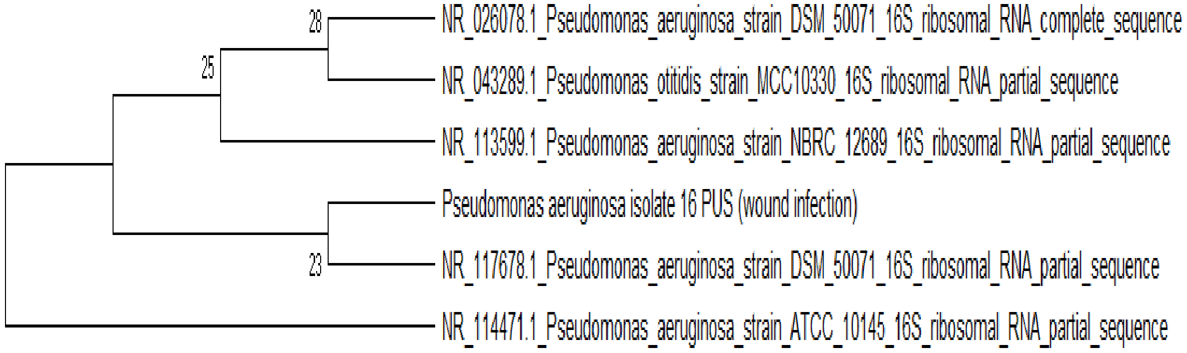

Fig. S2 Bootstrap tree for *P. aeruginosa* isolate (16) 16S ribosomal RNA sequence against 16S ribosomal RNA sequences retrieved from NCBI GenBank.

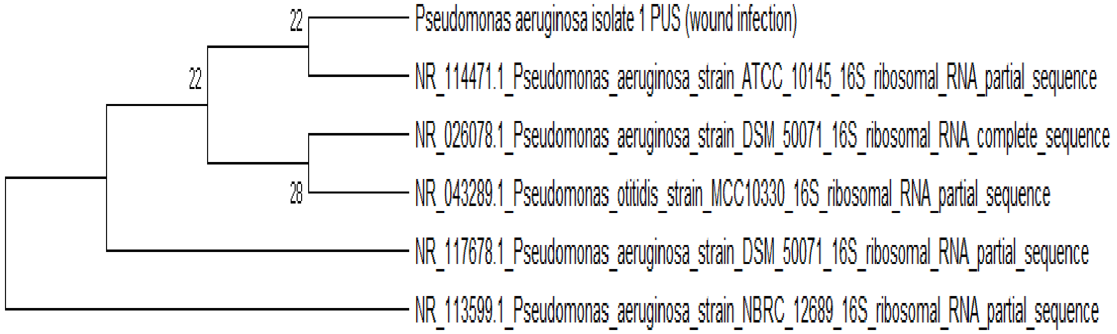

Fig. S3 Bootstrap tree for *P. aeruginosa* isolate (1) 16S ribosomal RNA sequence against 16S ribosomal RNA sequences retrieved from NCBI GenBank.

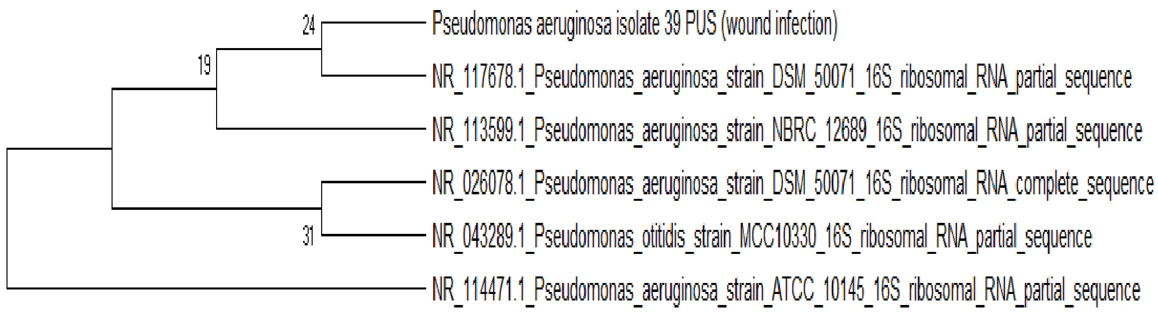

Fig. S4 Bootstrap tree for *P. aeruginosa* isolate (39) 16S ribosomal RNA sequence against 16S ribosomal RNA sequences retrieved from NCBI GenBank.

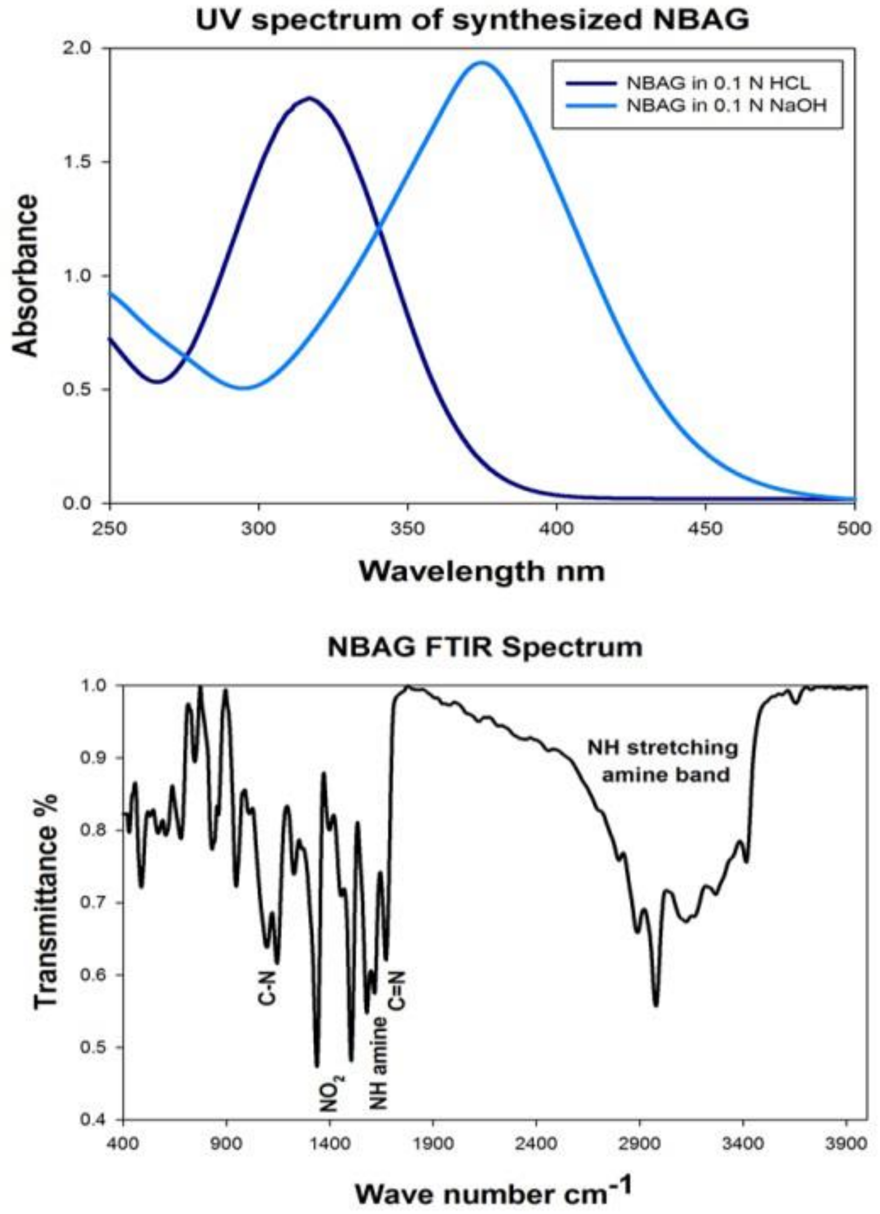

Fig. S5 UV and FTIR absorbance spectra for lyophilized powder nitrobenzylidine aminoguanidine (NBAG).

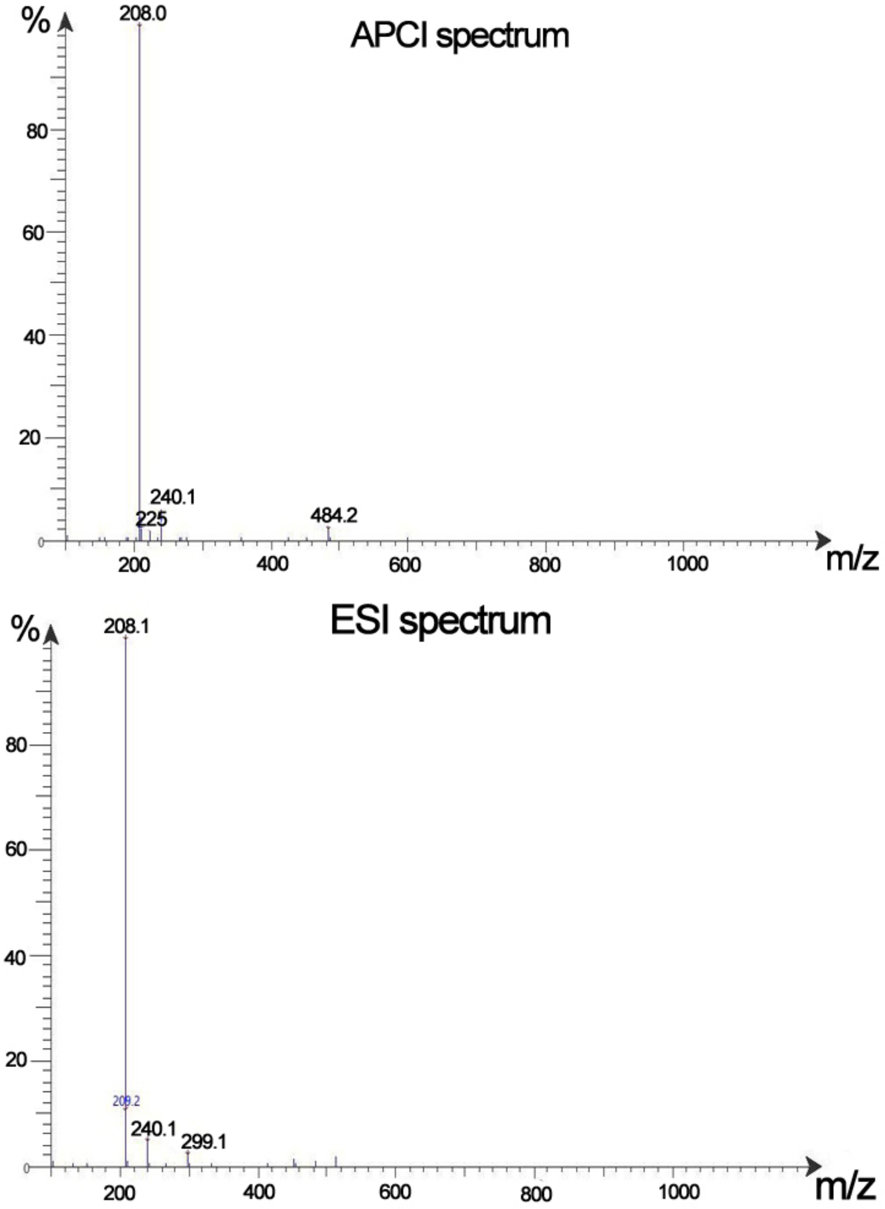

Fig. S6 Mass spectra for lyophilized powder, nitrobenzylidine aminoguanidine (NBAG)

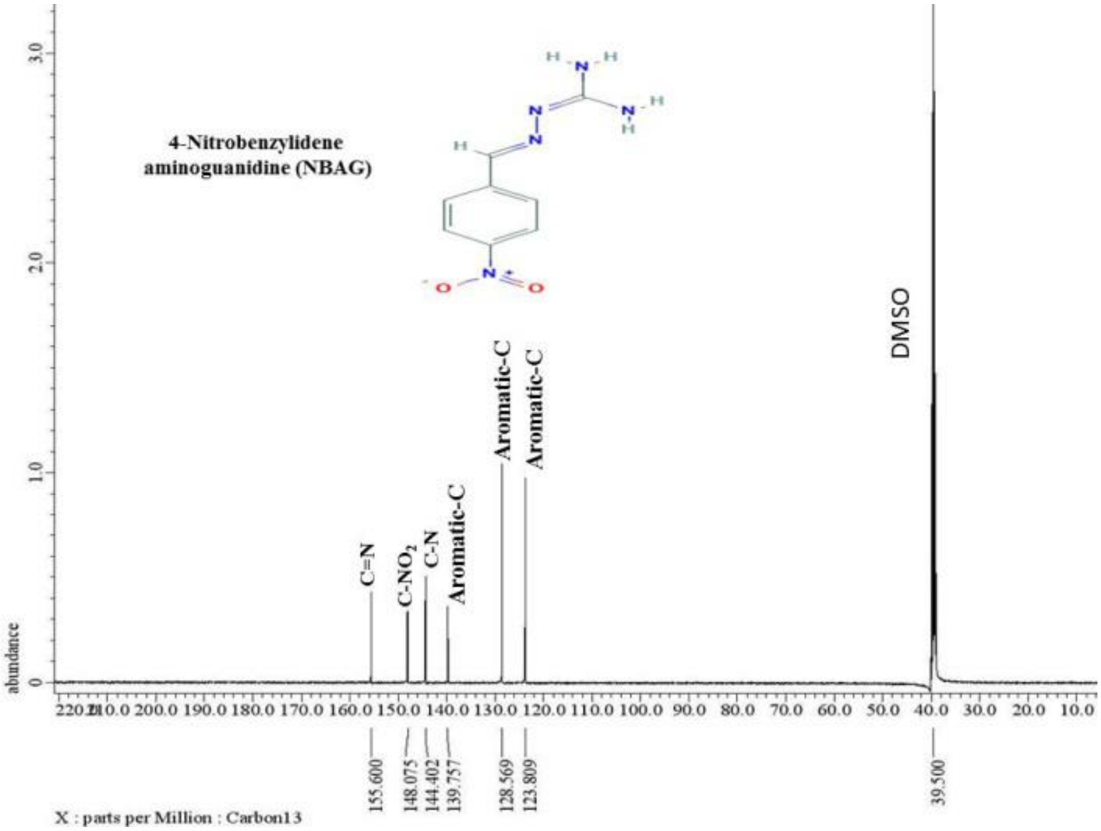

Fig. S7 ^{13}C-NMR spectrum for lyophilized powder, nitrobenzylidine aminoguanidine (NBAG).

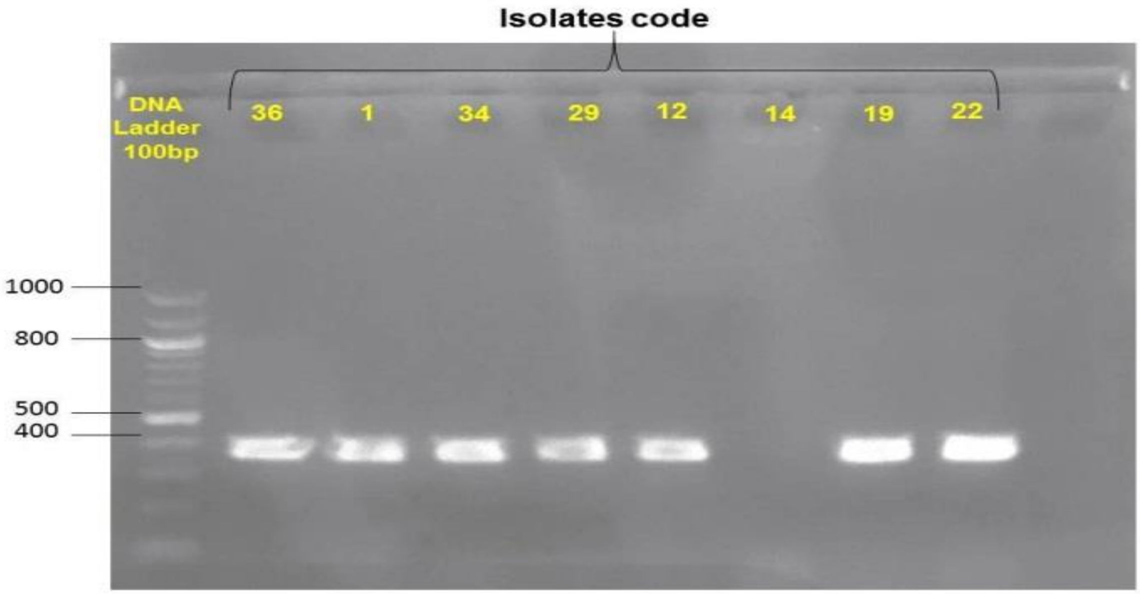

Fig. S8 Agarose Gel electrophoresis for the detection of *tox*A amplicons.

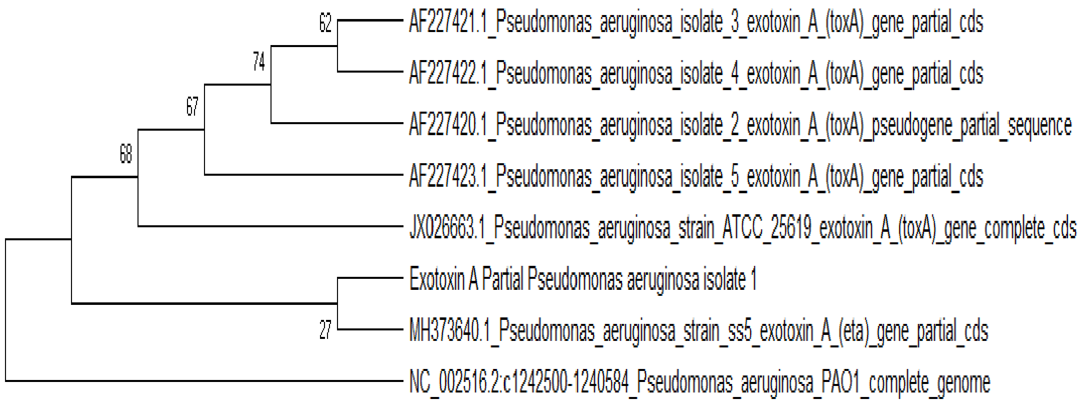

Fig. S9 Bootstrap tree for partial sequence of *tox*A gene (Exotoxin A Partial *P. aeruginosa* isolate 1) blasted against *tox*A gene records retrieved from NCBI GenBank.

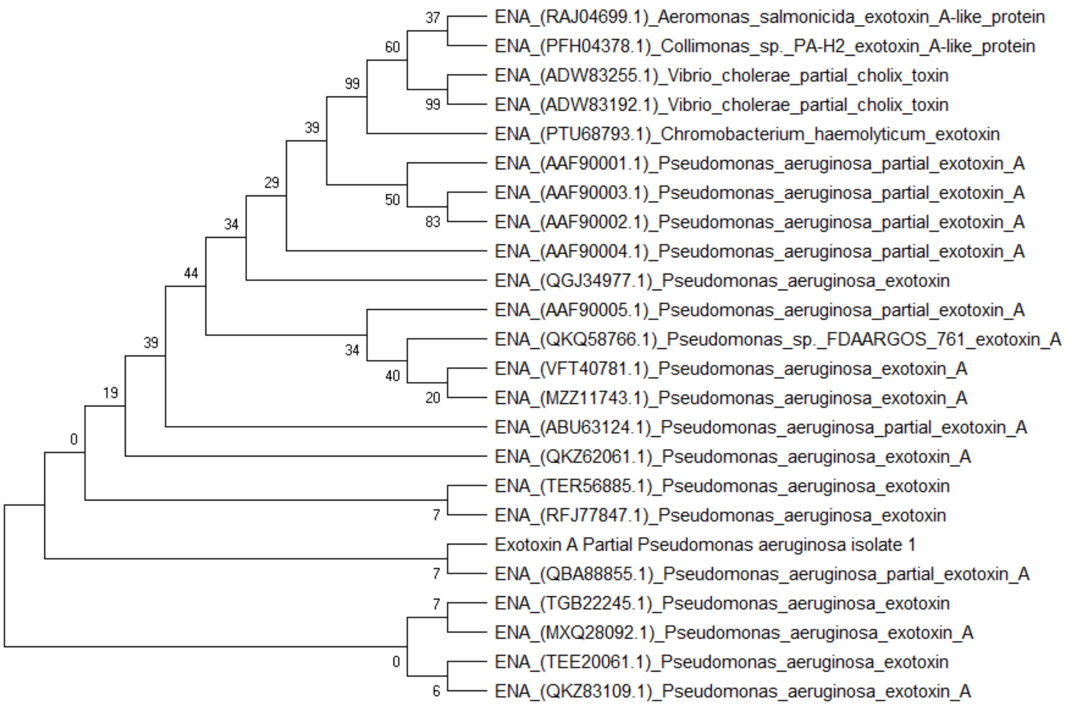

Fig S10. Bootstrap tree for partial sequence of *tox*A gene (Exotoxin A Partial *P. aeruginosa* isolate 1) blasted against *tox*A gene records retrieved from European nucleotide Archives (ENA).

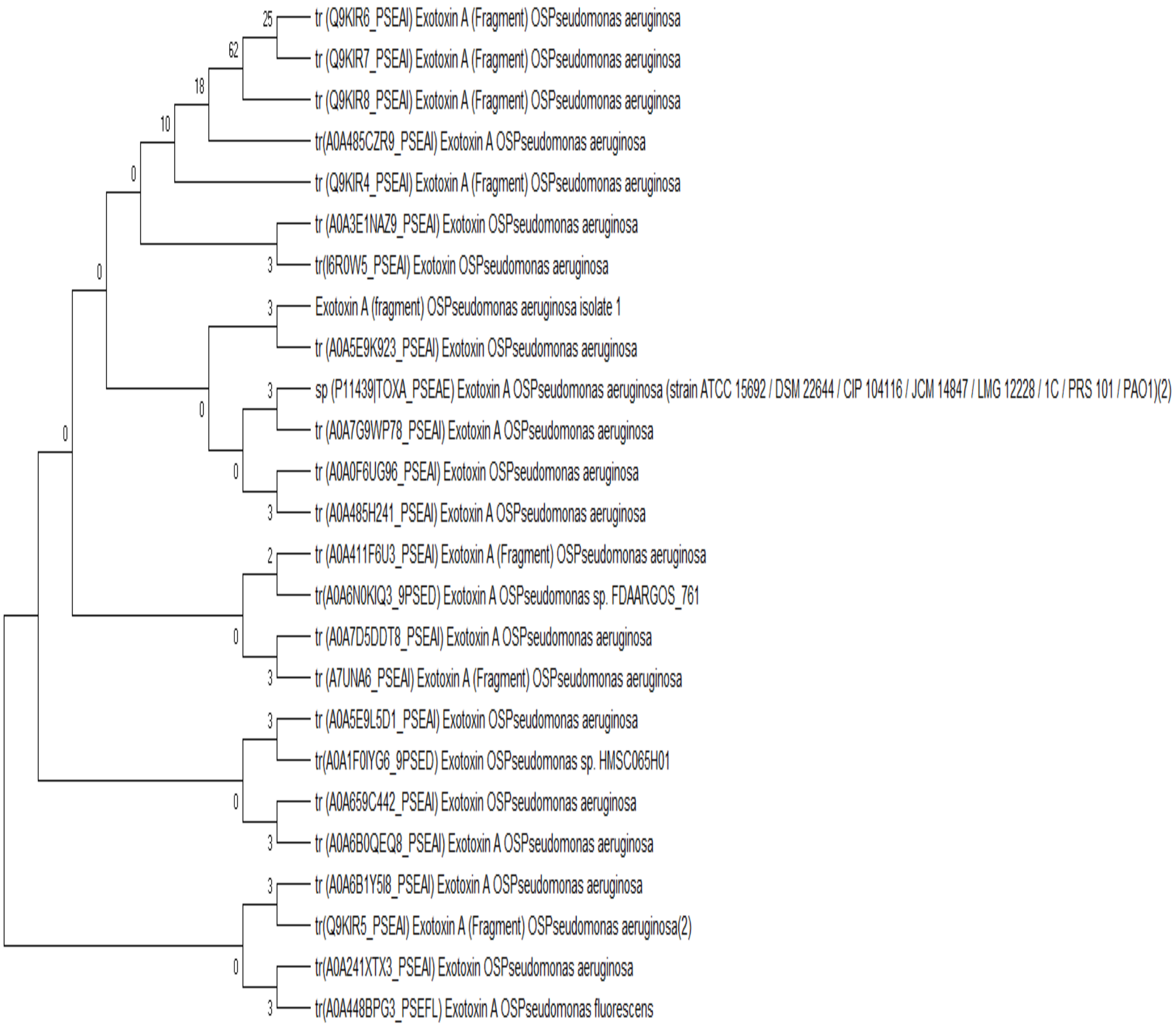

Fig. S11 Bootstrap tree for translated *tox*A gene (Exotoxin A (Fragment) OS *P. aeruginosa* isolate 1) blasted against exotoxin A protein records retrieved from Uniprot Database.

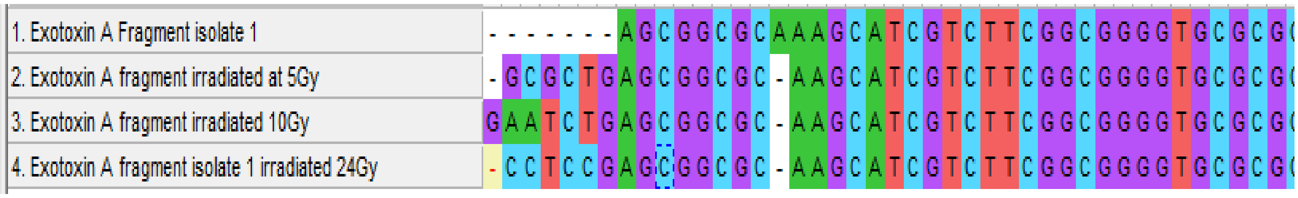

Fig. S12 Alignment of *tox*A sequence and deletion in the Adenine base

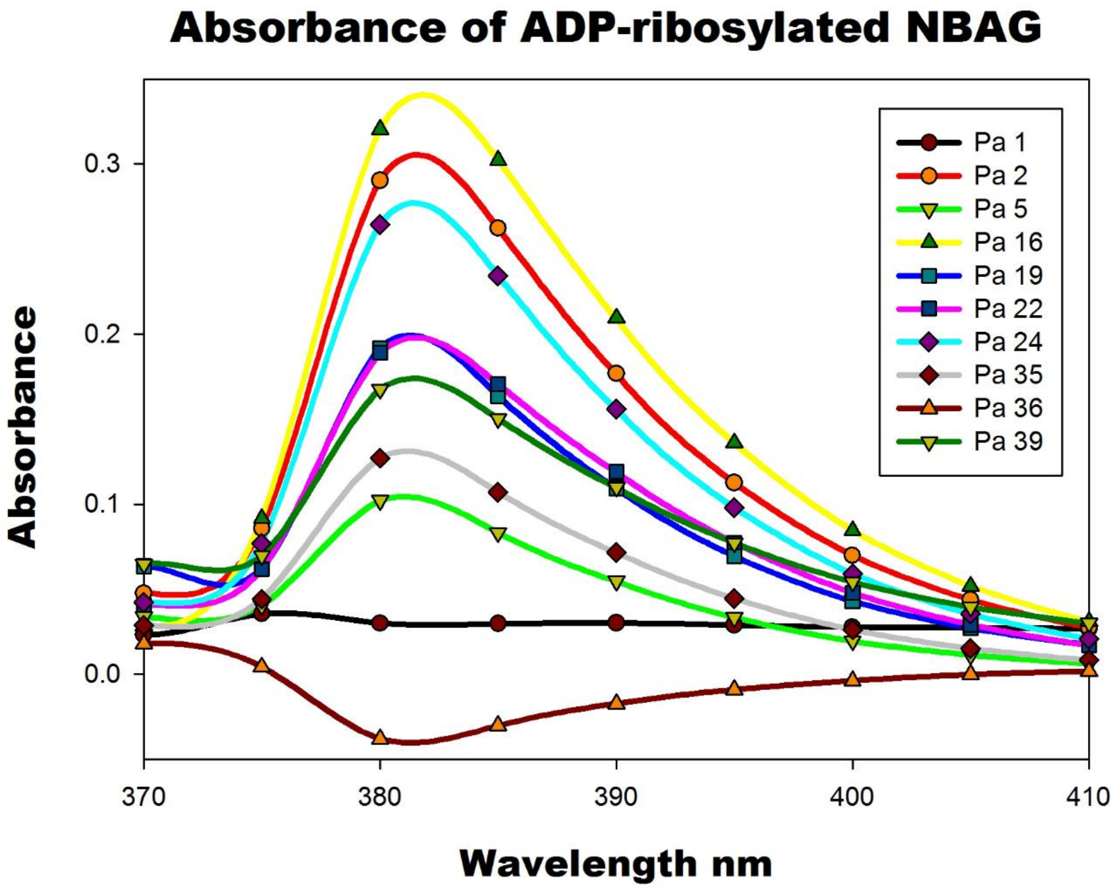

Fig. S13 The absorbance spectra of ADP ribosylated nitrobenzylidine aminoguanidine (NBAG).

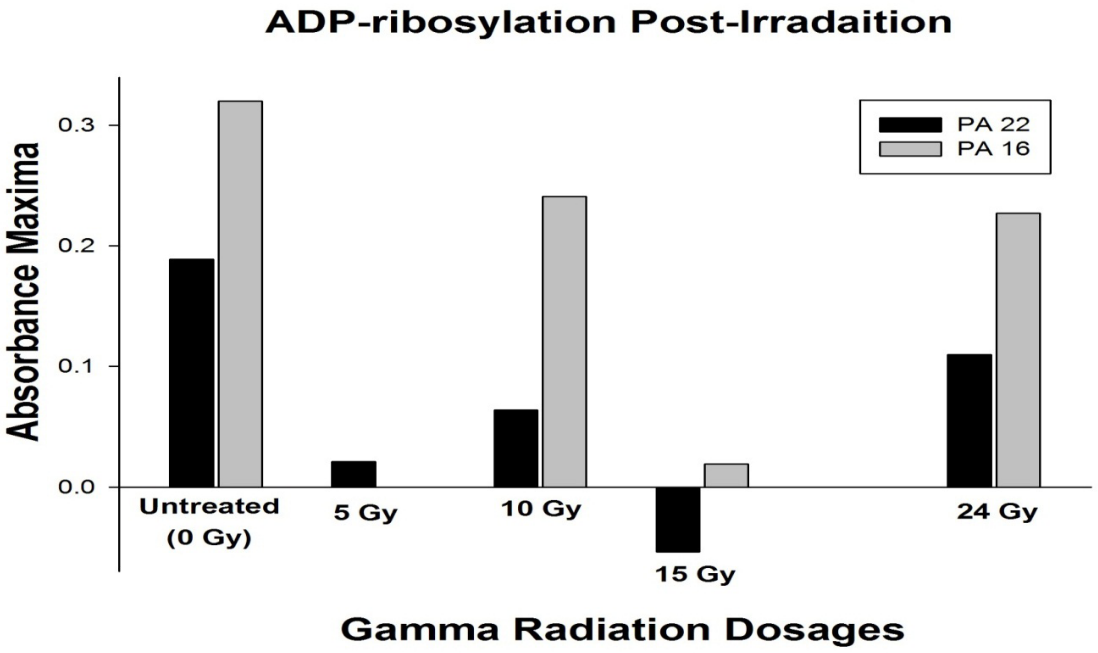

Fig. S14 Reduction in the absorbance maxima post exposure to gamma irradiated exotoxin A protein extract.

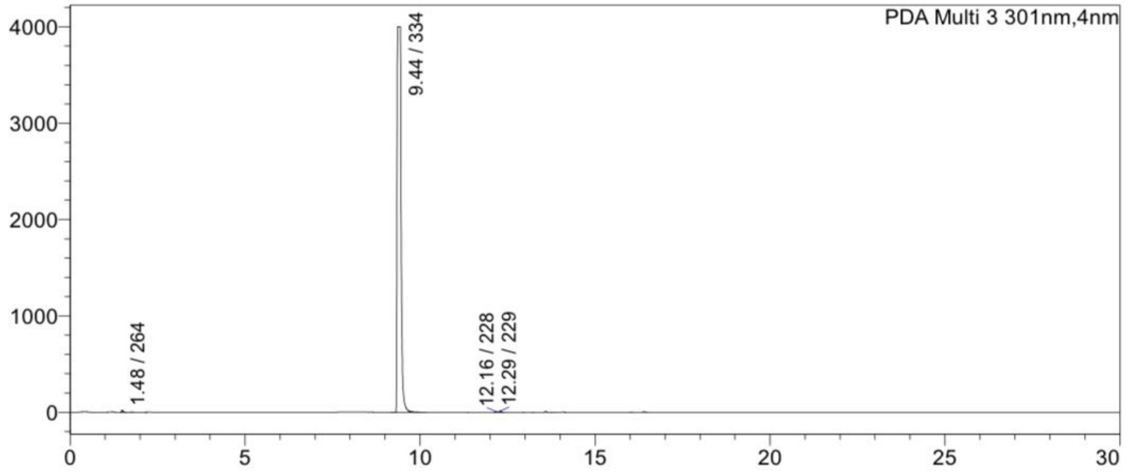

Fig. S15 HPLC chromatogram of *P. aeruginosa* isolate 1 at 301 nm.

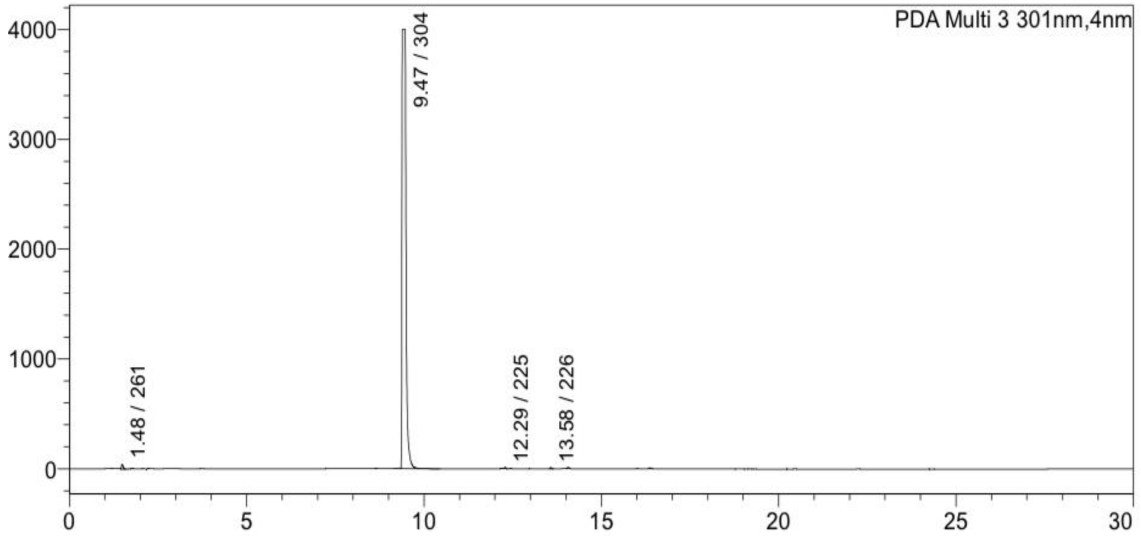

Fig. S16 HPLC chromatogram of *P. aeruginosa* isolate 5 at 301 nm**.**

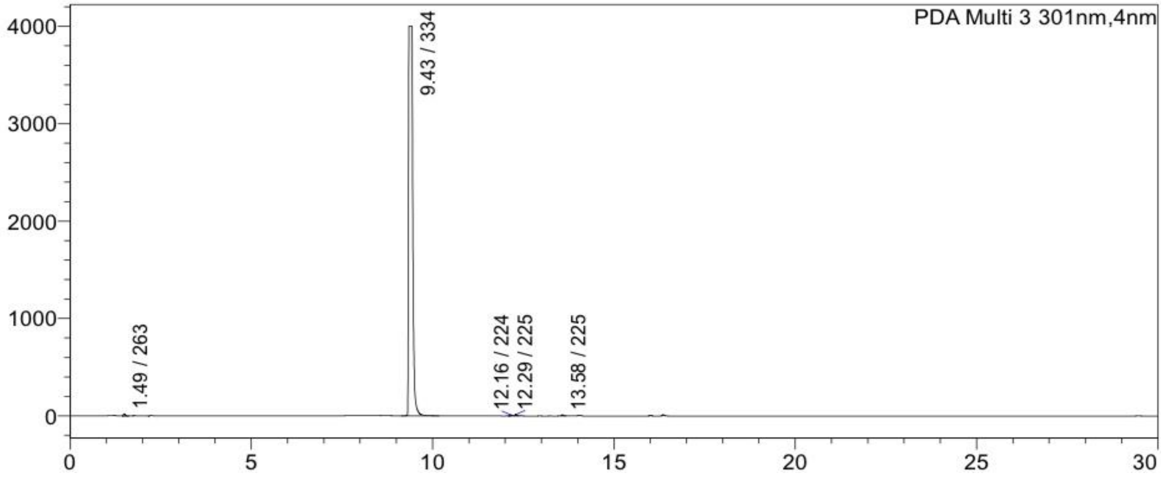

Fig. S17 HPLC chromatogram of *P. aeruginosa* isolate 35 at 301 nm.

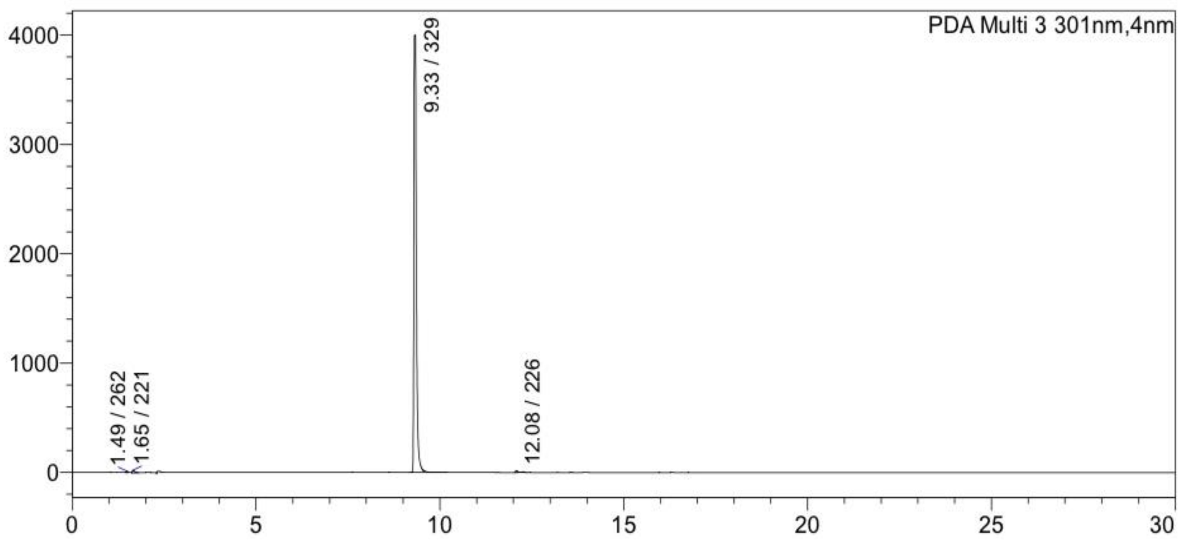

Fig. S18 HPLC chromatogram of PA 39 isolate at 301 nm.

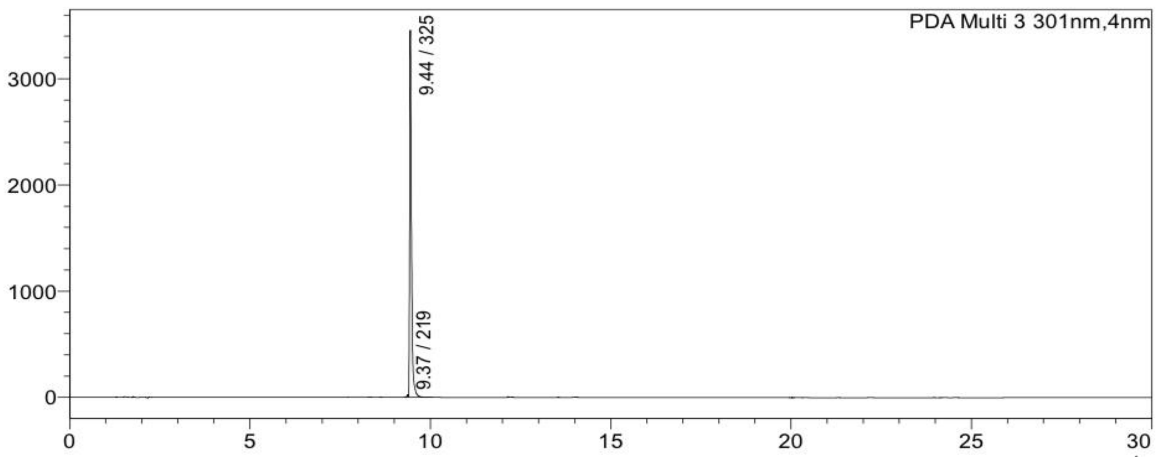

Fig. S19 HPLC chromatogram of *P. aeruginosa* isolate 22 irradiated at 5Gy at 301 nm.

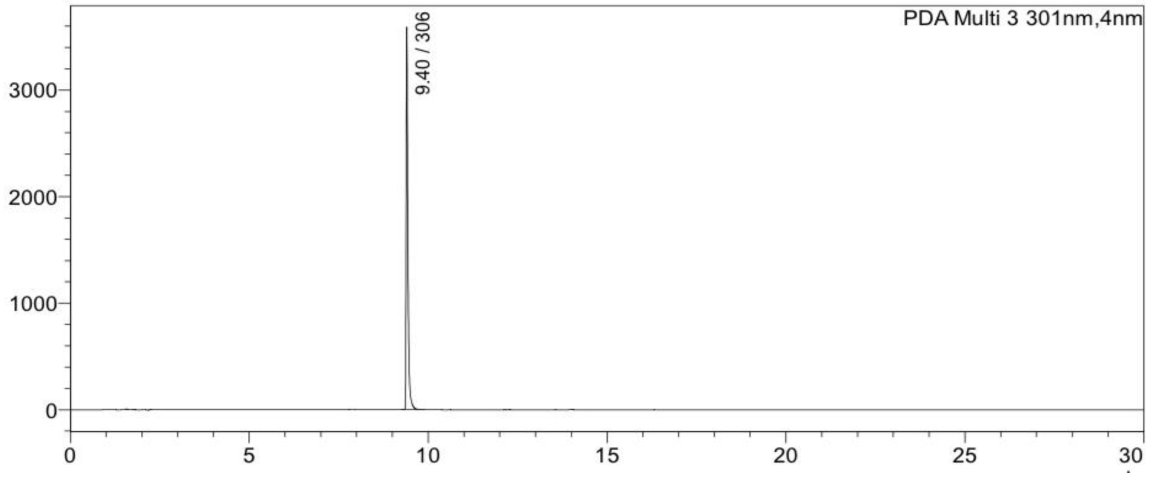

Fig. S20 HPLC chromatogram of *P. aeruginosa* isolate 22 irradiated at 15Gy at 301 nm**.**

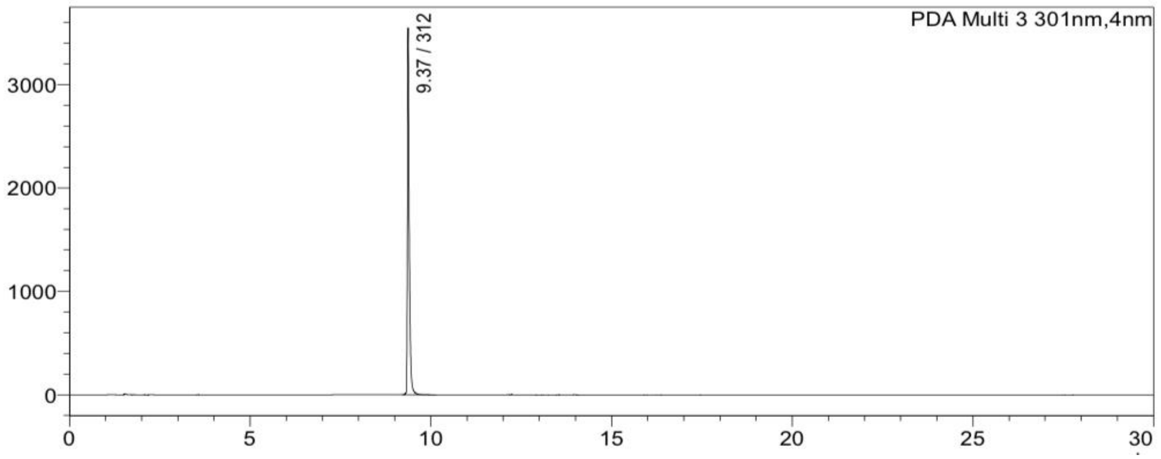

Fig. S21 HPLC chromatogram of *P. aeruginosa* isolate 22 irradiated at 10Gy at 301 nm.

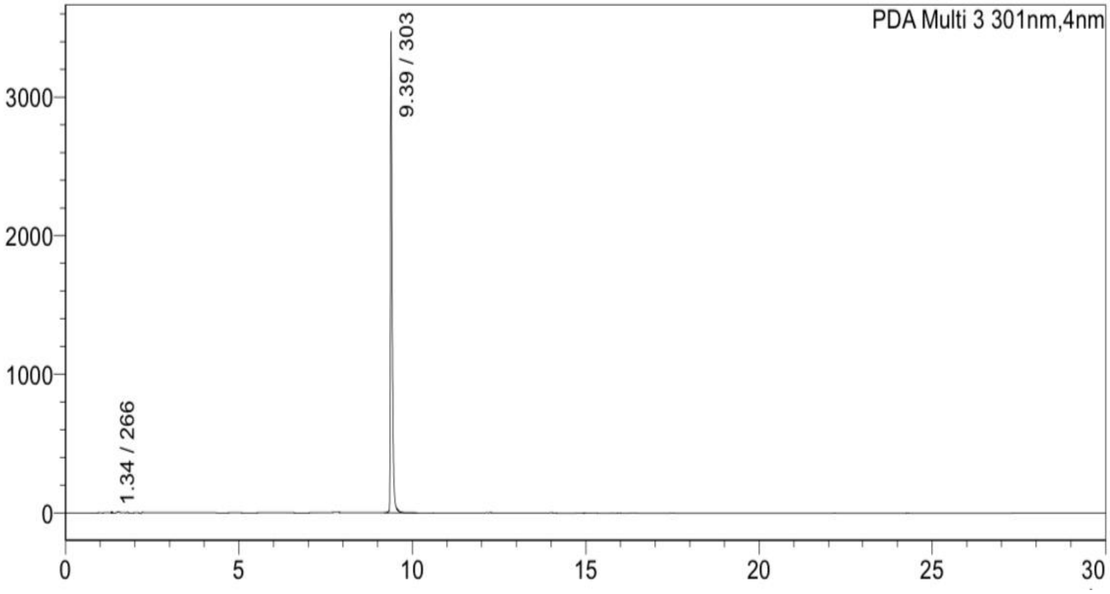

Fig. S22 HPLC chromatogram of *P. aeruginosa* isolate 16 irradiated at 15Gy at 301 nm**.**

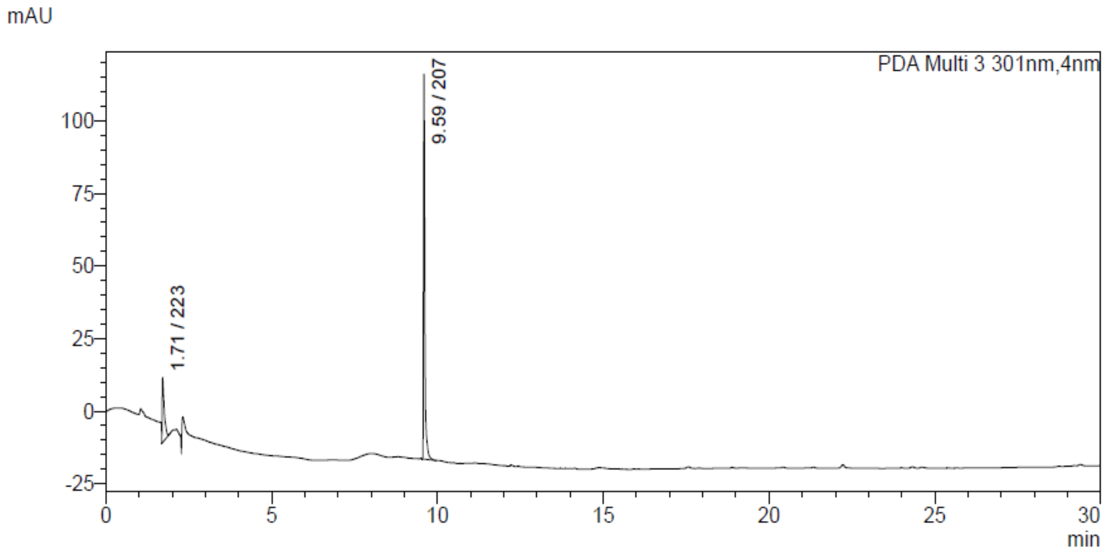

Fig. S23 HPLC chromatogram of *P. aeruginosa* isolate16 irradiated at 10Gy at 301 nm.

Table S1 Computed Pairwise distance between *P. aeruginosa* isolate 22, 16S ribosomal RNA and 16S ribosomal RNA sequences retrieved from NCBI GenBank.

P. aeruginosa isolate 22 PUS (Wound infection)					
NR_113599.1_*P. aeruginosa* strain NBRC 12689 16S ribosomal RNA partial sequence	0.0000000000				
NR_026078.1_*P. aeruginosa* strain DSM 50071 16S ribosomal RNA complete sequence	0.0155488761	0.0145572551			
NR_043289.1_ *Pseudomonas otitidis* strain MCC10330 16S ribosomal RNA partial sequence	0.0140164920	0.0138233270	0.0280647425		
NR_117678.1_*P. aeruginosa* strain DSM 50071 16S ribosomal RNA partial sequence	0.0000000000	0.0000000000	0.0152632412	0.0132377634	
NR_114471.1_*P. aeruginosa* strain ATCC 10145 16S ribosomal RNA partial sequence	0.0000000000	0.0000000000	0.0149673751	0.0149327869	0.0013455292

Table S2 Computed Pairwise distance between *P. aeruginosa* isolate 16, 16S ribosomal RNA and 16S ribosomal RNA sequences retrieved from NCBI GenBank.

P. aeruginosa isolate 16 PUS (wound infection)					
NR_113599.1_*P. aeruginosa* strain NBRC 12689 16S ribosomal RNA partial sequence	0.0000000000				
NR_026078.1_*P. aeruginosa* strain DSM 50071 16S ribosomal RNA complete sequence	0.0149986237	0.0145564497			
NR_043289.1_*Pseudomonas otitidis* strain MCC10330 16S ribosomal RNA partial sequence	0.0177800921	0.0138227115	0.0280619679		
NR_117678.1_*P. aeruginosa* strain DSM 50071 16S ribosomal RNA partial sequence	0.0000000000	0.0000000000	0.0152622684	0.0132371974	
NR_114471.1_P. aeruginosa strain ATCC 10145 16S ribosomal RNA partial sequence	0.0000000000	0.0000000000	0.0149665773	0.0149320673	0.0013455201

Table S3 Computed Pairwise distance between *P. aeruginosa* isolate 1, 16S ribosomal RNA and 16S ribosomal RNA sequences retrieved from NCBI GenBank.

P. aeruginosa isolate 1 PUS (wound infection)					
NR_026078.1_*P. aeruginosa* strain DSM 50071 16S ribosomal RNA complete sequence	0.07543700008				
NR_043289.1_*Pseudomonas otitidis* strain MCC10330 16S ribosomal RNA partial sequence	0.07521891720	0.02809952200			
NR_117678.1_*P. aeruginosa* strain DSM 50071 16S ribosomal RNA partial sequence	0.05845264710	0.01527665850	0.01324401410		
NR_114471.1_*P. aeruginosa* strain ATCC 10145 16S ribosomal RNA partial sequence	0.05534673080	0.01497816030	0.01494033330	0.00134562880	
NR_113599.1_P. aeruginosa strain NBRC 12689 16S ribosomal RNA partial sequence	0.05300979010	0.01456859140	0.01383011020	0.00000000000	0.00000000000

Table S4 Computed Pairwise distance between *P. aeruginosa* isolate 39, 16S ribosomal RNA and 16S ribosomal RNA sequences retrieved from NCBI GenBank.

P. aeruginosa isolate 39 PUS (wound infection)					
NR_113599.1_*P. aeruginosa* strain NBRC 12689 16S ribosomal RNA partial sequence	0.05333537340				
NR_026078.1_*P. aeruginosa* strain DSM 50071 16S ribosomal RNA complete sequence	0.06475885670	0.00997315810			
NR_043289.1_*Pseudomonas otitidis* strain MCC10330 16S ribosomal RNA partial sequence	0.06509173940	0.00945560900	0.01923748590		
NR_117678.1_P. aeruginosa strain DSM 50071 16S ribosomal RNA partial sequence	0.05333537340	0.00000000000	0.01045678320	0.00905477790	
NR_114471.1_*P. aeruginosa* strain ATCC 10145 16S ribosomal RNA partial sequence	0.05333537340	0.00000000000	0.01025144650	0.01021265210	0.00091946050

Table S5 Computed Pairwise distance between Exotoxin A partial sequence *P. aeruginosa* isolate 1 and *tox*A gene sequences retrieved from NCBI GenBank.

AF227420.1_*P. aeruginosa* isolate 2 exotoxin A (*tox*A) pseudogene partial sequence							
AF227421.1_*P. aeruginosa* isolate 3 exotoxin A (*tox*A) gene partial cds	0.0205743847						
AF227422.1_*P. aeruginosa* isolate 4 exotoxin A (*tox*A) gene partial cds	0.0275753798	0.0067221006					
AF227423.1_*P. aeruginosa* isolate 5 exotoxin A (*tox*A) gene partial cds	0.0312056578	0.0101408300	0.0169881228				
Exotoxin_A Partial *P. aeruginosa* isolate 1	0.0276264342	0.0135494470	0.0204285976	0.0101134303			
JX026663.1_*P. aeruginosa* strain ATCC 25619 exotoxin A (*tox*A) gene complete cds	0.0241215214	0.0101408300	0.0169881228	0.0067282737	0.0033510143		
MH373640.1_*P. aeruginosa* strain ss5 exotoxin A (eta) gene partial cds	0.0276264342	0.0135494470	0.0204285976	0.0101134303	0.0000000000	0.0033510143	
NC_002516.2:c1242500-1240584_*P. aeruginosa* PAO1 complete genome	0.0276264342	0.0135494470	0.0204285976	0.0101134303	0.0000000000	0.0033510143	0.0000000000

Table S6 Computed Pairwise distance between Exotoxin A partial sequence *P. aeruginosa* isolate 1 and *tox*A gene sequences retrieved from European nucleotide archive (ENA) and NCBI GenBank.

AF227420.1 *P. aeruginosa* isolate 2 exotoxin A (*tox*A) pseudogene partial sequence															
AF227421.1 *P. aeruginosa* isolate 3 exotoxin A (*tox*A) gene partial cds	0.0204563125														
AF227422.1_*P. aeruginosa* isolate 4 exotoxin A (*tox*A) gene partial cds	0.0274237076	0.0066793394													
AF227423.1_*P. aeruginosa* isolate 5 exotoxin A (*tox*A) gene partial cds	0.0310408693	0.0100783694	0.0168873948												
ENA_(AAF90001.1)_*P. aeruginosa* partial exotoxin_A	0.0239420977	0.0033323990	0.0100503275	0.0066978736											
ENA_(AAF90002.1) *P. aeruginosa* partial exotoxin A	0.0204563125	0.0000000000	0.0066793394	0.0100783694	0.0033323990										
ENA_(AAF90003.1)_*P. aeruginosa* partial exotoxin A	0.0274237076	0.0066793394	0.0000000000	0.0168873948	0.0100503275	0.0066793394									
ENA_(AAF90004.1)_*P. aeruginosa* partial exotoxin A	0.0310408693	0.0100783694	0.0168873948	0.0000000000	0.0066978736	0.0100783694	0.0168873948								
ENA_(AAF90005.1)_*P. aeruginosa* partial exotoxin A	0.0346351960	0.0203673208	0.0273037014	0.0168873948	0.0238375844	0.0203673208	0.0273037014	0.0168873948							
ENA_(TEE20061.1)_*P. aeruginosa* exotoxin	0.0274763337	0.0134674157	0.0203096466	0.0100503275	0.0168873948	0.0134674157	0.0203096466	0.0100503275	0.0134546939						
ENA_(TER56885.1)_*P. aeruginosa* exotoxin	0.0274763337	0.0134674157	0.0203096466	0.0100503275	0.0168873948	0.0134674157	0.0203096466	0.0100503275	0.0134546939	0.0000000000					
ENA_(TGB22245.1)_*P. aeruginosa* exotoxin	0.0274763337	0.0134674157	0.0203096466	0.0100503275	0.0168873948	0.0134674157	0.0203096466	0.0100503275	0.0134546939	0.0000000000	0.0000000000				
Exotoxin_A_Partial_*P. aeruginosa* isolate 1	0.0274763337	0.0134674157	0.0203096466	0.0100503275	0.0168873948	0.0134674157	0.0203096466	0.0100503275	0.0134546939	0.0000000000	0.0000000000	0.0000000000			
JX026663.1_*P. aeruginosa* strain ATCC 25619 exotoxin A (*tox*A) gene complete cds	0.0239877949	0.0100783694	0.0168873948	0.0066855966	0.0134801617	0.0100783694	0.0168873948	0.0066855966	0.0100688798	0.0033292931	0.0033292931	0.0033292931	0.0033292931		
MH373640.1_*P. aeruginosa* strain ss5 exotoxin A (eta) gene partial cds	0.0274763337	0.0134674157	0.0203096466	0.0100503275	0.0168873948	0.0134674157	0.0203096466	0.0100503275	0.0134546939	0.0000000000	0.0000000000	0.0000000000	0.0000000000	0.0033292931	
NC_002516.2:c1242500-1240584_*Pseudomonas_aeruginosa* PAO1 complete genome	0.0274763337	0.0134674157	0.0203096466	0.0100503275	0.0168873948	0.0134674157	0.0203096466	0.0100503275	0.0134546939	0.0000000000	0.0000000000	0.0000000000	0.0000000000	0.0033292931	0.0000000000

Table S7 Absorbance maxima and expected amount of ADP-ribosylated NBAG formed by *P. aeruginosa* exotoxin A protein extract.

***P. aeruginosa* strains (Code number of tested isolate)**	**Absorbance Maxima at ʎmax ≈380-385 nm**	**ADP ribosylated NBAG formed (µmol/ml)**	**Percentage of ADP ribosylated NABG%**
1	0.054	0.03	0.003
2	0.290	0.15	0.015
5	0.102	0.05	0.005
16	0.320	0.16	0.016
19	0.192	0.10	0.010
22	0.189	0.09	0.009
24	0.264	0.13	0.013
35	0.127	0.06	0.006
36	0.047	0.02	0.002
39	0.167	0.08	0.008

Absorbance maxima; highest absorbance in UV absorbance spectrum: ADP-ribosylated NBAG formed = (absorbance maxima x (5x10^{-2}))/0.1: Percentage of ADP-ribosylated NBAG% = (conc. of ADP ribosylated NBAG/ conc. of NBAG) x100

Table S8 Absorbance maxima post exposure to gamma irradiated exotoxin A extract and percent reduction in ADP-ribosylated NBAG formed.

**Gamma irradiated exotoxin A extract (Gy)**	**Absorbance Maxima at ʎmax ≈380**	**ADP-ribosylated NBAG formed (µmol/ml)**	**Reduction in ADP-ribosylated NBAG ***	**Percent reduction in ADP ribosylated NBAG****
P. aeruginosa isolate 22				
0 (untreated)	0.189	0.09		
5	0.021	0.01	-0.08	88.80
10	0.063	0.03	-0.06	66.19
15	-0.053		-	
24	0.110	0.06	-0.04	41.73
P. aeruginosa isolate 16				
0 (untreated)	0.320	0.16		
10	0.240	0.12	-0.04	24.77
15	0.019	0.01	-0.15	94.01
24	0.226	0.11	-0.05	29.14

ADP-ribosylated NBAG formed = (absorbance maxima x (5x10^{-2}))/0.1: * Reduction in the amount of ADP-ribosylated NBAG= conc. of ADP-ribosylated NBAG in irradiated samples – conc. of ADP-ribosylated NBAG in untreated samples: ** Percent reduction in the amount of ADP ribosylated NBAG = (reduction in conc. of ADP ribosylated NBAG/conc. of ADP-ribosylated NBAG in untreated samples) x 100

Table S9 Percent cytotoxicity and viability for exotoxin A protein extract on cultured *Hep2* cells.

P. aeruginosa strains (Code number)	% cytotoxicity incurred at 10 µg/ml	% cytotoxicity incurred at 100 µg/ml	% cell viability at 10 µg/ml	P value	% cell viability at 100 µg/ml	P value
1	23.03	96.43	76.97±0.51	$8.85x10^{-3}$	3.57 ±0.47	$1.87x10^{-4}$
5	11.55	99.02	88.45±0.41	$1.29x10^{-4}$	0.98 ±0.20	$6.13x10^{-5}$
16	22.56	99.11	77.44±1.51	$1.25x10^{-4}$	0.89 ±0.05	$5.33x10^{-6}$
22	38.13	99.18	61.87±0.11	$7.78x10^{-4}$	0.82 ±0.07	$6.13x10^{-6}$
24	11.83	99.63	88.17±1.22	$2.62x10^{-4}$	0.37 ±0.10	$1.16x10^{-5}$
35	9.40	97.02	90.60±0.53	$5.39x10^{-4}$	2.98 ±0.55	$1.76x10^{-4}$
36	13.23	98.99	86.77±0.49	$4.46x10^{-2}$	1.23 ±0.39	$1.56x10^{-4}$
39	26.50	99.20	73.55±0.40	$1.05x10^{-3}$	0.80 ±0.02	$2.12x10^{-6}$

%cytotoxicity = 100- ((mean OD treated/mean OD negative control) x100); % cell viability = 100-%cytotoxicity; ± Standard deviation (SD); P value ≤ 0.05 (significant). The test was carried out in 3 replicates, the positive control used was doxorubicin and negative control was tris buffer. The P value was calculated from the deviation of cell viability % post exposure to exotoxin A protein extract and positive control doxorubicin.

Table S10 Percent cell viability and increase in cellular viability post exposure to irradiated *P. aeruginosa* exotoxin A protein extract.

Radiation Dosage (Gy) (for *Pa* 22 strain)	% cell viability at 10 µg/ml	P value	%cell viability at 100 µg/ml	P value	% Increase in cell viability post exposure to gamma irradiated protein at 10 µg/ml	%Increase in cell viability post exposure to gamma irradiated protein extract at 100 µg/ml	IC50 µg/ml
0	61.87±0.11	$7.78x10^{-4}$	0.82±0.07	$6.13x10^{-6}$			15.3
5	84.61±0.84	$1.56x10^{-5}$	4.73±0.45	$3.04x10^{-6}$	22.74	3.91	23.2
10	68.64±1.41	$1.34x10^{-4}$	4.55±0.28	$6.60x10^{-7}$	6.77	3.73	16.19
15	63.94±2.45	$2.91x10^{-4}$	7.47±0.85	$1.14x10^{-5}$	2.07	6.65	32.71
24	77.29±1.56	$5.71x10^{-5}$	1.11±0.45	$2.7x10^{-6}$	15.42	0.29	17.01

Pa: *Pseudomonas aeruginosa*; %cytotoxicity = 100- ((mean OD treated/mean OD negative control) x100); % cell viability = 100-%cytotoxicity; % increase in cell viability post exposure to gamma radiation = %cell viability at specified radiation dose - % cell viability without exposure to gamma radiation; ; ± Standard deviation (SD); P value ≤ 0.05 (significant). The test was carried out in 3 replicates, the positive control used was doxorubicin and negative control was tris buffer. The P value was calculated from the deviation of cell viability % post exposure to irradiated exotoxin A protein extract and untreated exotoxin A extract.
